# Advances in the understanding of nuclear pore complexes in human diseases

**DOI:** 10.1007/s00432-024-05881-5

**Published:** 2024-07-30

**Authors:** Yuxuan Li, Jie Zhu, Fengguang Zhai, Lili Kong, Hong Li, Xiaofeng Jin

**Affiliations:** 1 The Affiliated Lihuili Hospital of Ningbo University, Ningbo, 315040 Zhejiang China; 2 Department of Biochemistry and Molecular Biology, and Zhejiang Key Laboratory of Pathophysiology, Health Science Center, Nngbo University, Ningbo, 315211 Zhejiang China

**Keywords:** Nuclear pore complexes, Nucleoporin, Cancers, Diseases

## Abstract

**Background:**

Nuclear pore complexes (NPCs) are sophisticated and dynamic protein structures that straddle the nuclear envelope and act as gatekeepers for transporting molecules between the nucleus and the cytoplasm. NPCs comprise up to 30 different proteins known as nucleoporins (NUPs). However, a growing body of research has suggested that NPCs play important roles in gene regulation, viral infections, cancer, mitosis, genetic diseases, kidney diseases, immune system diseases, and degenerative neurological and muscular pathologies.

**Purpose:**

In this review, we introduce the structure and function of NPCs. Then We described the physiological and pathological effects of each component of NPCs which provide a direction for future clinical applications.

**Methods:**

The literatures from PubMed have been reviewed for this article.

**Conclusion:**

This review summarizes current studies on the implications of NPCs in human physiology and pathology, highlighting the mechanistic underpinnings of NPC-associated diseases.

## Introduction

The nucleus is a membrane-bound organelle in eukaryotic cells that safeguards and preserves genetic material and coordinates vital cellular processes (Dey and Baum 2021). There are several components of the nucleus (I). Nuclear envelope: the nuclear envelope (NE) is composed of two lipid bilayers, namely the inner and outer membranes (INM and ONM), which are separated by a perinuclear space. The NE also contains nuclear pore complexes (NPCs) that penetrate the double membrane (Dey and Baum 2021; Schwarz and Blower 2016; Lin and Hoelz 2019), and the whole structure of the NE encloses the nucleus and separates the contents from the cytoplasm. NPCs in the NE allow the selective transport of molecules between the nucleus and cytoplasm (Lin and Hoelz 2019). Notably, the NE is not only a smooth outer boundary but is also interrupted by invagination, which reaches deep within the nucleoplasm and can traverse the nucleus completely (Malhas et al. 2011). (II). Nucleoplasm: the nucleoplasm is a gel-like matrix but is not homogenous within the nucleus, where various nuclear components are suspended (Galganski et al. 2017; Mancini et al. 1996). The nucleoplasm includes numerous types of nuclear bodies, also called nuclear domains or sub-compartments (Galganski et al. 2017). (III). Chromatin: chromatin is a complex of DNA and proteins that make up chromosomes. It consists of DNA wrapped around histone proteins to form nucleosomes, which condense to form higher-order structures. Chromatin is crucial for the regulation of gene expression, DNA replication, and repair.

Previous studies have shown that nuclear membrane proteins are involved in the pathogenesis of human diseases via transcriptional regulation, nuclear positioning, mechanotransduction, and signaling pathways. Morphological changes in the size and shape of the nucleus are widespread in cancer cells; however, little is known about the underlying molecular mechanisms and functional correlations. Hence, the relationship between NE and cancer is complex and multifaceted. For example, human NPCs serve as a gateway for the exchange of molecules between the nucleus and cytoplasm, including RNA molecules, proteins, and other macromolecules (Beck and Hurt 2017). Furthermore, many tumor suppressor proteins such as p53, breast cancer type 1 susceptibility protein (BRCA1), and phosphatase and tensin homolog (PTEN) rely on human NPCs for transport between the nucleus and cytoplasm (Ikliptikawati et al. 2023; Thompson 2010; Xie et al. 2021a). However, disruption of nucleocytoplasmic shuttling of these tumor suppressors can impair their normal functions, allowing uncontrolled cell growth and promoting tumor development. For instance, high expression levels of human nucleoporin NUP153, which facilitates nucleocytoplasmic transport, occur in hepatocellular carcinoma (HCC), probably leading to alterations in NPC composition and function (Gan et al. 2022; Nofrini et al. 2016). In addition, mutations in the human nucleoporin NUP98, which provides vital interaction sites for nucleocytoplasmic transport, have been found in hematologic malignancies, including leukemia, and contribute to the fusion of oncoproteins (Nofrini et al. 2016; McNeer et al. 2019; Chandra et al. 2022). Moreover, the human nucleoporins POM121 and importinβ mediate the nuclear import of a series of oncogenic transcription factors (e.g., such as the transcription factor E2F1 (E2F1), transcription factor p64 (c-Myc), and androgen receptor (AR), promoting the progression of prostate cancer (PCa) (Rodriguez-Bravo et al. 2018). Therefore, this review focuses on the link between nucleoporins in NPCs and diseases.

## The composition of NPCs

**Fig. 1:**
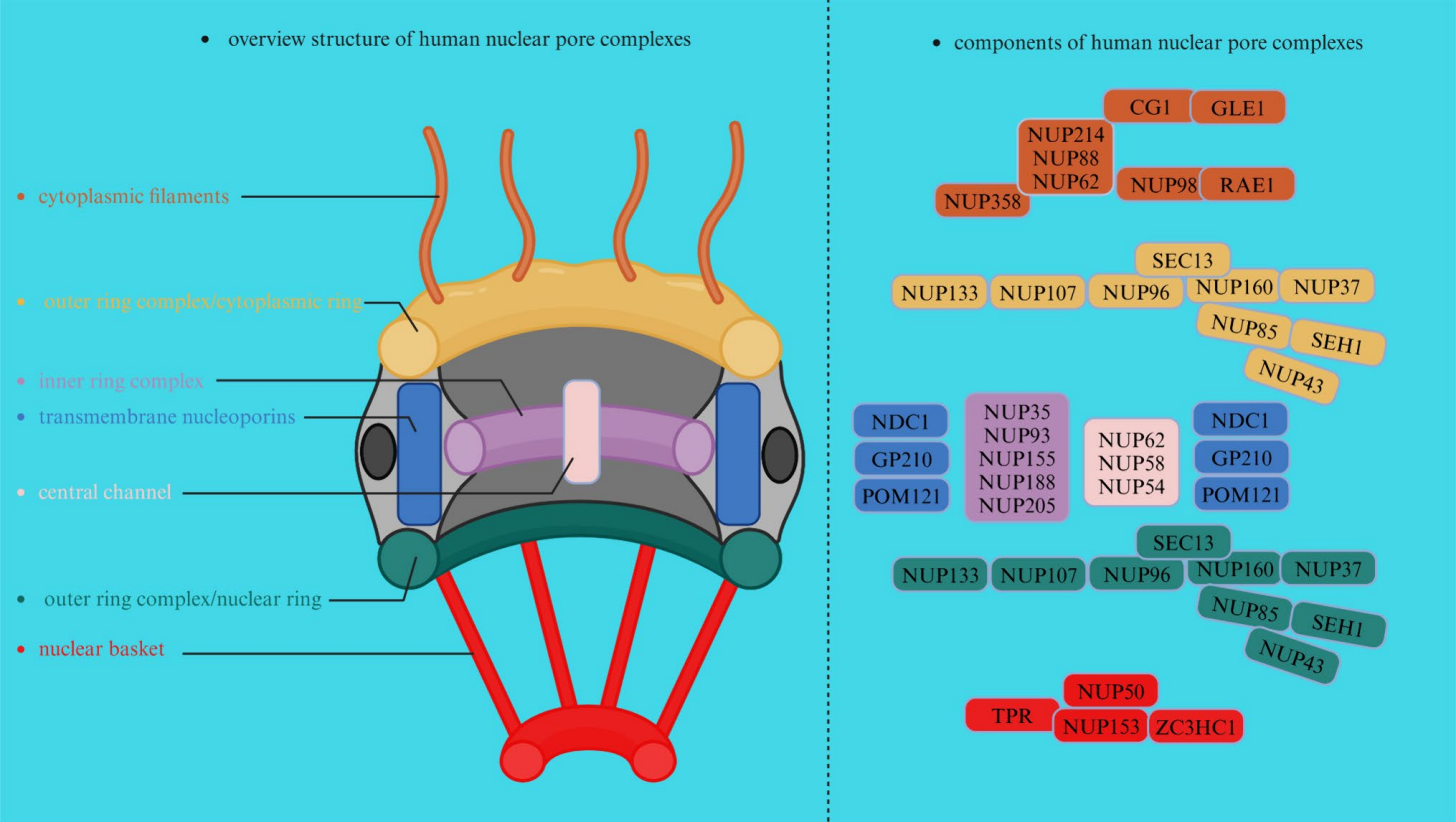
Human NPCs comprise seven parts, including cytoplasmic filaments, the outer ring complex (cytoplasmic rings and nuclear ring), transmembrane nucleoporins, the central channel, the inner ring complex, and the nuclear basket. On the left is a description of the general structure of the NPCs, and on the right is a description of the specific NUPs of each component

At the junction of the INM and ONM of the human nucleus, approximately 600 copies of approximately 30 proteins termed nucleoporins (NUPs) assemble into NPCs, with structural elements of stacked a-helical repeats and/or b-propellers; approximately one-third of these proteins also contain phenylalanine-glycin (FG) repeat sequences for the selective transport of cargoes (Nofrini et al. 2016). These NUPs assemble into distinct subcomplexes within the NPCs, contributing to their overall organization and function. Generally, NPCs are classified based on their composition and substructure: (I). Outer ring complex: the outer ring complex, including the cytoplasmic ring (CR) and nuclear ring (NR), which are anchored by the inner pore ring, forms the structural framework of the NPCs (Beck and Hurt 2017). It consists of several NUPs, including NUP93, NUP205, NUP188, and NUP155, among others (Nofrini et al. 2016), forming a circular structure at the periphery of the NPCs and serving as a docking site for other nucleoporin subcomplexes. (II). Inner ring complex: the inner ring complex containing NUPs such as NUP58, NUP54, and NUP62, among others, is located on the nuclear side of the NPCs and connects the outer ring complex to the central channel, helping anchor the nuclear basket and contributing to the selective permeability of the NPCs (Nofrini et al. 2016; Kosinski et al. 2016; Gaik et al. 2015). (III). Central channel: the central channel of NPCs forms the main transport gate for molecules, such as proteins, RNA, and ribosomal subunits, passing shuttling between the nucleus and cytoplasm, and consists of NUPs, including NUP62, NUP58, and NUP54 (Beck and Hurt 2017). (IV). Nuclear basket: the nuclear basket is a specialized structure within NPCs that extends into the nucleus, where it plays a role in the anchoring and organization of NPCs, as well as in the regulation of nucleocytoplasmic transport (Kosinski et al. 2016; Gaik et al. 2015; Cibulka et al. 2022). The nuclear basket contains NUPs such as NUP153, NUP50, zinc finger C3HC-type protein 1 (ZC3HC1), and translocated promoter region protein (TPR) (Beck and Hurt 2017; Appen et al. 2015; Gunkel et al. 2021). (V). Cytoplasmic filaments: cytoplasmic filaments containing several NUPs, such as NUP358, NUP214, NUP98, NUP88, ALADIN, mastermind-like domain-containing protein 1 (CG1), and the mRNA export factor RAE1 (RAE1), contribute to the anchoring of NPCs to the NE and are involved in interactions with cytoplasmic factors associated with transport regulation (Lin and Hoelz 2019; Hamada et al. 2011; Wälde et al. 2012). Specifically, cytoplasmic filaments are peripheral elements emanating from the nucleus and CR, and are extensions of NPCs that project into the cytoplasm (Beck and Hurt 2017; Nofrini et al. 2016). Cytoplasmic filament NUPs recruit cargo transport factor complexes for nucleocytoplasmic transport and orchestrate the export and remodeling of messenger ribonucleoprotein particles in preparation for translation (Bley et al. 2022). (VI). Transmembrane NUPs POM121, nuclear pore membrane glycoprotein 210 (GP210), and nuclear division cycle protein 1 (NDC1) enclose the central channel and play a gatekeeping role, and their stability affects the levels of several other NUPs (Coyne et al. 2020; Liu et al. 2009; Gomez-Cavazos and Hetzer 2015) (Fig. 1).

## Outer ring complex

The CR contains 16 copies of the Y-shaped complex (Y-complex), encircling head to tail to form the inner and outer layers of eight Y-complexes (Fontana et al. 2022; Knockenhauer and Schwartz 2016; Kelley et al. 2015). The Y-complex is composed of a short arm (Nup160 and Nup37), long arm (Nup85, Nup43, and Seh1), and stem (Nup96, Sec13, Nup107, and Nup133) in Xenopus laevis (Fontana et al. 2022; Bilokapic and Schwartz 2012). Previous studies have shown that Seh1 and Nup43, although indispensable in mouse embryonic stem cells, are required for their average cell growth rates, viability upon differentiation, and maintenance of proper NPC density (Gonzalez-Estevez et al. 2021). In addition, Nup133, including TPR and Nup153, is required for proper nuclear basket assembly and dynamics in mouse embryonic stem cells (Souquet et al. 2018; Orniacki et al. 2023). Moreover, in Xenopus laevis oocytes, the CR contains two copies of Nup205, two copies of the Nup214-Nup88-Nup62 complex, one Nup155, and five copies of Nup358 (Fontana et al. 2022). Significantly, Nup358 contains FG repeats previously shown to form a gel-like condensate phase for selective cargo passage (Knockenhauer and Schwartz 2016; Frey et al. 2006; Lemke 2016). Additionally, four Nup358 copies clamp around the Y-complexes to stabilize CR (Fontana et al. 2022). Under the anchoring effect of Nup358, there are eight cytoplasmic filaments, namely, Nup88, Nup214, Nup98, the mRNA export factor GLE1 (GLE1), CG1, ribonucleic acid export 1 (RAE1), and Nup62 (Beck and Hurt 2017; Sakiyama et al. 2016, 2017).

### NUP160 and NUP37

**Fig. 2:**
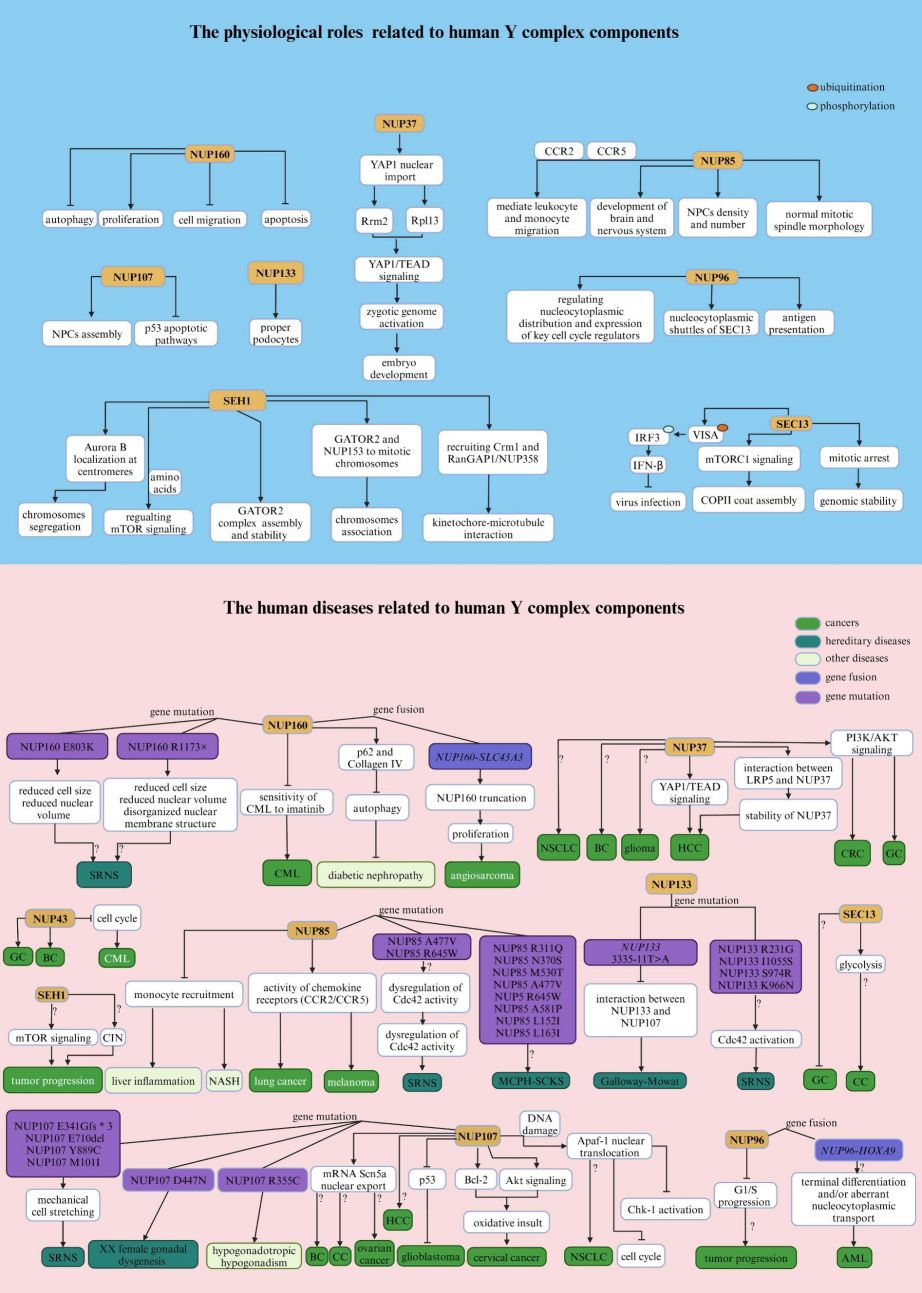
Summary of the physiological and pathological effects of each component of the Y complex in humans

In Xenopus laevis, full-length Nup160 consists of a seven-bladed β-propeller followed by an extended α-helical domain, and its C-terminal fragment is located at the vertex of the Y complex and sandwiched between Sec13 homolog 1 (Seh1) and SEC13-like protein 1 (Sec13) (Zhu et al. 2022). A previous study showed that Nup160 interacts with Nup98 and Nup153 and plays a role in mRNA export but not in protein import or export in Xenopus laevis (Vasu et al. 2001). Functionally, *Nup160* knockdown inhibits cell proliferation; induces apoptosis, autophagy, and cell migration; and alters the expression and localization of podocyte-associated molecules in mouse podocytes (Xie et al. 2021b). Phenotypically, silencing of Drosophila *Nup160* in nephrocytes (fly renal cells) led to functional abnormalities, reduced nuclear volume and cell size, and a disorganized nuclear membrane structure (NPC and nuclear lamin localization defects) (Zhao et al. 2019). NUP160 was found to be highly expressed in the peripheral blood or cell lines of patients with chronic myeloid leukemia (CML), inhibiting the sensitivity of CML to imatinib (Zhang et al. 2019). In human angiosarcoma, the frequent gene fusion *NUP160-SLC43A3* causes truncation of *NUP160* and stimulates cell proliferation (Shimozono et al. 2015). In addition to *NUP160* mutations caused by gene fusion, compound heterozygous mutations in *NUP160* (R1173× and E803K) have been reported in steroid-resistant nephrotic syndrome (SRNS) (Zhao et al. 2019). Notably, although Nup160 is initially associated with nucleoplasmic transport, it is also involved in autophagy, hybrid sterility, female sterility, abnormal morphology, and slow development in flies (Xie et al. 2021b; Maehara et al. 2012) (Fig. 2).

Human NUP37, also known as P37 or MCPH24, is vital for kinetochore-microtubule interactions and mitosis (Loïodice et al. 2004). Recent bioanalysis studies have suggested that NUP37 is a potential prognostic biomarker and oncogene for non-small cell lung cancer (NSCLC), breast cancer (BC), glioma, and pan-cancer (He et al. 2022; Li and Liu 2021; Liu et al. 2021a). Additionally, tumor tissues with increased NUP37 expression exist in a relatively immunosuppressive microenvironment and are resistant to multiple anticancer drugs (He et al. 2022). Previous studies have shown that increased NUP37 expression in HCC acts as a positive regulator of Yes-associated protein 1/TEA domain family member (YAP1/TEAD) signaling, thereby promoting cancer progression (Luo et al. 2017). Similarly, maternal NUP37 contributes to the nuclear import of YAP1 and subsequently activates YAP1/TEAD signaling, which is involved in oocyte maturation and preimplantation embryo development in mammals (Peng et al. 2022; Guo et al. 2022). Moreover, the interaction between NUP37 and low-density lipoprotein-related receptor 5 (LRP5) enhances the stabilization of NUP37, thereby facilitating liver cancer (LC) cell proliferation (Chen et al. 2019). However, LRP5 is mainly expressed in cell membranes, suggesting that LRP5 is likely to enter the nucleus and interact with NUP37 in the nucleus (Chen et al. 2019). Interestingly, the epigenetic mechanism of elevated NUP37 expression in HCC is closely associated with the aberrant hypomethylation pattern of the NUP37 promoter (cg24826236, cg08316365, and cg08085165) (Tang et al. 2022a, b). In colorectal cancer (CRC) cell lines, upregulated expression of NUP37 promotes the activation of phosphatidylinositol-3-kinase/protein kinase B (PI3K/AKT) signaling, thereby facilitating the progression of CRC (Xiong et al. 2023). Similarly, overexpression of NUP37 activates PI3K/AKT signaling, promoting the proliferation, migration, and invasion of gastric cancer (GC) cells (Zhang et al. 2021a), and forms an immunosuppressive microenvironment in gliomas (He et al. 2022) (Fig. 2** and **Table 1). Notably, several vital proteins involved in PI3K/AKT signaling, including AKT, mechanistic target of rapamycin complex 1 (mTORC1), PTEN, and 3-phosphoinositide-dependent kinase 1 (PDK1), can shuttle between the nucleus and cytoplasm (Chen et al. 2022; Gupta et al. 2022). However, the relationship between NUP37 and these proteins in terms of nucleoplasmic transport remains unclear.

**Table 1:** Related human diseases and pathological functions of each component of the Y complex

Nucleoporins	Diseases	Abnormal changes	Functions	References
NUP160	CML	Upregulation	Inhibiting the sensitivity of CML cells to imatinib	Zhang et al. (2019)
	Angiosarcoma	Gene fusion (NUP*160-SLC43A3*)	Promoting cell proliferation	Shimozono et al. (2015)
	SRNS	Gene mutation (R1173 × and E803K)	Function loss, reduced cell size and nuclear volume, and disorganized NE structure	Zhao et al. (2019)
NUP37	Glioma	Upregulation	Resistant to anticancer drugs Promoting cell proliferation	He et al. (2022), Liu et al. (2021a)
	BC	Upregulation	Promoting cell proliferation, migration, and stemness	Li and Liu (2021)
	HCC	Upregulation	Activation of YAP/TEAD signaling Promoting cell proliferation, migration, and invasion	Luo et al. (2017), Chen et al. (2019)
	CRC	Upregulation	Activation of PI3K/AKT signaling Promoting cell proliferation, migration, and invasion Inhibiting cell apoptosis and cell cycle arrest	Xiong et al. (2023)
	GC	Upregulation	Activation of PI3K/AKT signaling Promoting cell proliferation, migration, and invasion	Zhang et al. (2021a)
NUP85	SRNS	Gene mutation (A477V and R645W)	ND	Braun et al. (2018a)
	MCPH–SCKS	gene mutation (R311Q, N370S, M530T, A477V, R645W, A581P, L152I and L163I)	ND	Ravindran et al. (2021, 2023)
	lung cancer	ND	Activation of chemokine (CCR2/5) Promoting tumor progression and decreases macrophage tumor-promoting activity	Terashima et al. (2020)
	melanoma	ND	Activation of chemokine (CCR2/5) Promoting tumor progression and decreases macrophage tumor-promoting activity	Terashima et al. (2020)
	NASH	ND	Inhibiting liver inflammation	Zhang et al. (2023)
NUP43	GC	Upregulation	Promoting cell malignant phenotype	Li et al. (2020), Yang et al. (2022)
	BC	Upregulation	Promoting DNA amplification	Ren et al. (2022), Tian et al. (2018), Zhang et al. (2021b), Jiang et al. (2021)
	CML	Upregulation	Inhibiting proliferative potential and cell cycle progression	Liu et al. (2019)
NUP96	AML	gene fusion (*NUP96-HOXA9*)	ND	Nakamura et al. (1996), Borrow et al. (1996)
SEC13	GC	ND	ND	Hussein et al. (2022)
	CC	ND	ND	Liu et al. (2022)
NUP107	SRNS	Gene mutation (M101I, E341Gfs*, E710del and Y889C)	ND	Braun et al. (2018b)
	XX female gonadal dysgenesis	Gene mutation (D447N)	ND	Weinberg-Shukron et al. (2015)
	Hypogonadotropic hypogonadism	Gene mutation (R355C)	ND	Ren et al. (2018)
	Glioblastoma	Upregulation	Promoting p53 degradation and tumorigenesis Inhibiting cell apoptosis	Ikliptikawati et al. (2023), Banerjee et al. (2010)
	Cervical cancer	Upregulation	Promoting expression of Bcl-2 and nucleocytoplasmic transport factors Activation of Akt signaling Resistance to oxidative damage	Shi et al. (2020)
	HCC	Upregulation	ND	Nong et al. (2023)
NUP133	Galloway-Mowat syndrome	Gene mutation (c.3335-11T>A)	Function loss Microcephaly, fewer neuronal cells, underdeveloped glomeruli, and fusion of the foot processes of the podocytes	Fujita et al. (2018)
	SRNS	Gene mutation (R231G, I1055S, S974R and K966N)	ND	Braun et al. (2018a), Wang et al. (2023a)

### NUP85, NUP43, and SEH1

In terms of biological function, human NUP85 (also known as FROUNT) plays a significant role in the development of tissues, particularly those of the brain and nervous system (Ravindran et al. 2021, 2023). In addition, NUP85 is a cytosolic regulator that mediates leukocyte and monocyte migration by binding to the chemokine receptor CC chemokine receptor 2/5 (CCR2/5) (Toda et al. 2014; Esaki et al. 2014), and it is worth noting that the interaction between NUP85 and these two chemokine receptors can be blocked by disulfiram (Terashima et al. 2020). Crucially, CCR2/5 are major chemotactic regulators of tumor-promoting macrophages at the tumor site, and blocking these receptors could impede tumor progression in experimental animal models (Zhang et al. 2013; Sanford et al. 2013; Popivanova et al. 2009) (Fig. 2).

Initially, Nup85 was associated with childhood-onset SRNS in four affected individuals with intellectual disabilities in Xenopus (Braun et al. 2018a). Recently, biallelic and heterozygous human *NUP85* variants were found in primary autosomal recessive microcephaly and Seckel syndrome spectrum disorders (MCPH–SCKS) (Ravindran et al. 2021, 2023). However, to date, mutations in NUP85 have not been found in cancer studies. *NUP85* deficiency remarkably inhibits tumor progression in lung carcinoma and melanoma and decreases macrophage tumor-promoting activity by directly mediating chemokine receptor activity (Terashima et al. 2020). Similarly, blockade of Nup85 attenuates steatosis and monocyte recruitment caused by macrophage chaperone-mediated autophagy deficiency in nonalcoholic steatohepatitis (NASH) mice (Zhang et al. 2023). approximately 10–20% of NASH patients may progress to liver fibrosis, cirrhosis, and even LC (Kessoku et al. 2021), indicating that NUP85 is likely associated with the early tumorigenesis of LC.

Human NUP43 harbors a canonical β-propeller fold consisting of seven 40 repeats of Trp-Asp (WD) (13–73, 77–116,134–168, 175–212, 220–257, 264–344, and 353–38), and each WD40 repeat of NUP43 is composed of four antiparallel β-strands (Xu et al. 2015a). In addition, an ordered loop at the bottom surface of NUP43 WD40 may act as a bridge that directly binds to NUP85–SEH1 (Xu et al. 2015a). Spatially, NUP43 binds to NUP85 through its bottom surface and approaches NUP37 (Bui et al. 2013). The carcinogenic role of NUP43 has been confirmed in GC (Li et al. 2020; Yang et al. 2022) and BC (Ren et al. 2022; Tian et al. 2018; Zhang et al. 2021b; Jiang et al. 2021). In GC cells, the long noncoding RNA (lncRNA) NCK1-AS1 functions as a sponge for microRNA-137 (miR-137) to increase NUP43 expression, thereby contributing to GC malignant behaviors (Li et al. 2020). In addition, NUP43 was found to be abnormally highly expressed in luminal A and human epidermal growth factor receptor 2 (HER2)+ BC patients, and might independently predict poor prognosis in cancer patients (Tian et al. 2018). However, the upregulated expression of NUP43, which is targeted by the miR-409 gene, reduces the proliferative potential and inhibits cell cycle progression in CML cells (Liu et al. 2019) (Fig. 2, Table 1).

Human SEH1 is required for chromosome association (e.g., targeting GATOR2 and NUP153 to mitotic chromosomes), chromosome segregation (e.g., controlling Aurora B localization at centromeres), proper kinetochore function (e.g., stabilizing kinetochore‒microtubule interactions by recruiting chromosome region maintenance 1 (CRM1) and RanGTPase AP1 (RanGAP1)–NUP358), and efficient localization of the chromosomal passenger complex (Platani et al. 2009, 2018; Zuccolo et al. 2007). Additionally, SEH1 is a critical component of the GATOR2 complex (consisting of MIOS, WDR24, WDR59, SEH1, and SEC13), which acts as a switch for mTORC1 signaling depending on nutrient levels (Valenstein et al. 2022). In addition, the process of tumor metabolic reprogramming is frequently accompanied by upregulation of mTOR signaling, thereby facilitating enhanced nutrient acquisition for the production of metabolites essential for sustaining growth and proliferation (Szwed et al. 2021) (Fig. 2).

### NUP96, SEC13, NUP107, and NUP133

Nup96 and Nup98 are encoded by the same gene, *Nup98–Nup96*, which is a protein precursor and is produced during autoproteolysis, and both proteins are associated with mRNA export in yeast and mammals (Fontoura et al. 1999; Enninga et al. 2002; Faria et al. 2006). Interestingly, the upstream region of human *NUP96* is the *NUP98* coding region, and *NUP98* chromosomal translocation (fusion with the *Homeobox A9* (*HOXA9*) gene) has been observed in patients with acute myeloid leukemia (AML) (Nakamura et al. 1996; Borrow et al. 1996). Conceivably, gene fusion disrupts the function of NUP96, which requires NUP98-dependent autocatalytic processing from the NUP98-96 precursor protein to be properly localized and available (Fontoura et al. 1999). In addition, human NUP96 differentially regulates the nucleocytoplasmic distribution and expression of key cell cycle regulators of mRNAs/proteins (e.g., Cyclin B1, Cyclin D3, and CDK6) in a cell cycle-dependent manner (Chakraborty et al. 2008). Phenotypically, deficits in Nup96 in mice lead to slightly enhanced proliferation of T cells and bone marrow-derived macrophages, suggesting a potential role for Nup96 as a haploinsufficient tumor suppressor (Chakraborty et al. 2008). However, another study showed that deficits in Nup96 in mice impaired antigen presentation and T-cell proliferation upon immunization (Faria et al. 2006), suggesting that Nup96 functions exhibit organizational differences. Moreover, human NUP96 stably interacts with the WD repeat region of SEC13 (another nucleoporin associated with the formation of coat protein complex II (COPII)-coated vesicles) during interphase, mediating the shuttling of SEC13 and likely coupling functions between the nucleus and cytoplasm (Enninga et al. 2003) (Fig. 2).

Several studies have shown that human SEC13 mainly plays roles in two distinct complexes, including a component of the NPCs and the GATOR2 complex (Beck and Hurt 2017; Valenstein et al. 2022). Functionally, SEC13 also primarily regulates the metaphase/anaphase transition and contributes to maintaining genomic stability during mitosis (Sihn et al. 2005); thus, SEC13 can maintain gene stability or impact cell growth by regulating the corresponding signaling pathways, reflecting its potential opposite effect on cancers. Furthermore, in mice, proper myelination and remyelination require Sec13-mediated autocrine pleiotrophin signaling and protein transport via Sec13 (Liu et al. 2022). In addition, SEC13 is involved in the VISA-mediated antiviral signaling pathway by increasing VISA aggregation and ubiquitination, thus enhancing the phosphorylation and dimerization of interferon regulatory factor 3 (IRF3) to facilitate IFN-β production and strengthen antiviral immune activity (Chen et al. 2018a). In GC patients, the expression of SEC13 is significantly decreased and negatively correlated with tumor stage, suggesting that SEC13 may act as a tumor suppressor in GC (Hussein et al. 2022). However, the gene and protein expression of SEC13 is dramatically increased in colon cancer (CC) patients, which may be related to its glycolytic function, suggesting that SEC13 has opposite effects in different cancer backgrounds (Liu et al. 2022). Notably, glycolysis is highly active in tumor cells and its reprogramming is closely related to tumor occurrence and progression (Pavlova and Thompson 2016) (Fig. 2, Table 1).

Human NUP107, located in the core scaffold of NPCs, is characterized by a leucine zipper motif and a large number of kinase consistency sites in its carboxy-terminal region but does not contain FG repeats (Banerjee et al. 2010). During cell mitosis, NUP107 plays a crucial role in driving the assembly of NPCs (Zuccolo et al. 2007). In glioblastoma, NUP107 functions in transport surveillance by tethering proteasome 26 s-mediated p53 degeneration, thereby promoting tumorigenesis (Ikliptikawati et al. 2023). In addition, depletion of a single nucleoporin, NUP107, induces apoptosis in human astrocytoma cells (Banerjee et al. 2010). Furthermore, overexpression of NUP107 confers resistance to oxidative stress but does not affect the migration or proliferation of cervical cancer cells (Shi et al. 2020). In terms of transport, apoptotic protease activating factor-1 (Apaf-1), an adaptor protein critically involved in mitochondrial cell death, binds to the central domain of Nup107 (457–618) through its CED-4 domain, leading to the nuclear accumulation of Apaf-1 under DNA damage in mouse (Jagot-Lacoussiere et al. 2015). Interestingly, the nuclear presence of Apaf-1 is a positive prognostic factor in NSCLC patients (Zermati et al. 2007; Besse et al. 2004). In rat, Nup107 also facilitates the nuclear export of *sodium channel protein type 5 subunit alpha* (*Scn5a*) mRNA and consequently induces the protein expression of Scn5a, regulating cardiac electrophysiology (Scn5a‐encoded INa channel) (Guan et al. 2019). Increased Scn5a expression is associated with more aggressive tumor characteristics (e.g., BC, CC, and ovarian cancer), including lymph node invasion, recurrence of metastasis, and reduced survival (Brackenbury 2012; Luiz and Wood 2016). In HCC cells, NUP107 is upregulated, portends poor prognosis, and can predict the survival of patients with HCC with reasonable accuracy (Nong et al. 2023). Moreover, a single nucleotide variant in *NUP107* may be predictive of sensitivity to platinum chemotherapy in patients with ovarian cancer (Alanee et al. 2017). These findings imply that abnormal NUP107 expression is closely associated with tumor occurrence and progression. In addition, there is a strong association between childhood SRNS (dysfunction of podocytes is a hallmark of SRNS) and a high incidence of *NUP107* mutations (Braun et al. 2018b; Rosti et al. 2017; Miyake et al. 2015; Park et al. 2017). These *NUP107* mutations (c.303G>A, p.M101I; c.1021dupG, p.E341Gfs*3; c.2129_2131delAAG, p.E710del; c.2666A>G, p.Y889C) cause changes in the amino acid sequence of this gene, indicating that the function and structure of the NUP107 protein may change in SRNS (Braun et al. 2018b). However, the mechanism by which* NUP107* mutations cause the glomerular phenotype in humans remains unknown. Previous studies have shown that NPCs are closely related to mechanical transduction signals (Swift and Discher 2014; Fedorchak et al. 2014), and mechanical stretching reduces podocyte proliferation and cell volume by reorganizing the actin cytoskeleton *in vitro* (Endlich et al. 2001; Petermann et al. 2002). Hence, increased postnatal capillary pressure may be more prone to damaging podocytes with *NUP107* mutations, leading to mechanical cell stretching. *NUP107* missense mutations (c.1063C>T, p.R355C) have also been found in hypogonadotropic hypogonadism, and are an important cause of morbidity (Ren et al. 2018). Similarly, a recessive missense mutation of NUP107 (c.1339G> a, p.D447N) occurs in XX female gonadal dysgenesis, characterized by underdeveloped, dysfunctional ovaries (Weinberg-Shukron et al. 2015). The impact of Drosophila Nup107 p.D364N (corresponding to human NUP107 p.D447N) in Drosophila females resulted in almost complete sterility, with a marked reduction in progeny, morphologically aberrant eggshells, and disintegrating egg chambers (Weinberg-Shukron et al. 2015). These results indicate a pivotal role of NUP107 in female reproductive development and more common conditions, such as premature ovarian failure (Fig. 2, Table 1).

Previous physiological studies have revealed that Nup133 is essential for embryonic development but not for mouse embryonic stem cell proliferation (Souquet et al. 2018). Similarly, deficiency or dysfunction of Nup133 impairs NPC assembly, maternal transcription factor (TF) nuclear transport, and zygotic genome activation, whereas Nup133 overexpression promotes these processes during zebrafish early embryogenesis (Shen et al. 2022). Furthermore, *Nup133* deficiency impairs murine neural lineage differentiation (Lupu et al. 2008). Consistently, a *Nup133*-knockdown zebrafish model exhibited microcephaly, fewer neuronal cells, underdeveloped glomeruli, and fusion of the foot processes of podocytes (Fujita et al. 2018; Rogg et al. 2022). Pathologically, the human homozygous *NUP133* mutation c.3335-11T>A results in the insertion of 9 bp of the intronic sequence between exons 25 and 26 in the mutant transcript, impairing the interaction between NUP133 and NUP107 and thus causing Galloway–Mowat syndrome (Fujita et al. 2018). In SRNS, these* NUP133* mutations (c.691C>G, p.R231G; c.3164T>C, p.I1055S; c.2922T>G, p.S974R; c.182+387T>G; c.2898G>C, p.K966N) have been reported (Braun et al. 2018a; Wang et al. 2023a). Notably, there were no significant gene expression changes in Nup133-mutated podocytes, essentially excluding a direct loss- or gain-of-function mechanism, but the localization of NUP133 and NUP107 to the NE was mildly defective, leading to spreading defects (Rogg et al. 2022). This evidence suggests that this mild damage may only affect sensitive cell types, and changes in the levels of NUP133 protein may be the main consequence of the mutant variant. Indeed, a susceptible and critical signaling pathway in podocytes is small RhoGTPase signaling, and activation of small RhoGTPase cell division control protein 42 (Cdc42) is a common consequence of *NUP133* deficiency (Braun et al. 2018a; Rogg et al. 2022) (Fig. 2, Table 1).

### NUP214, NUP88 and NUP62

**Fig. 3:**
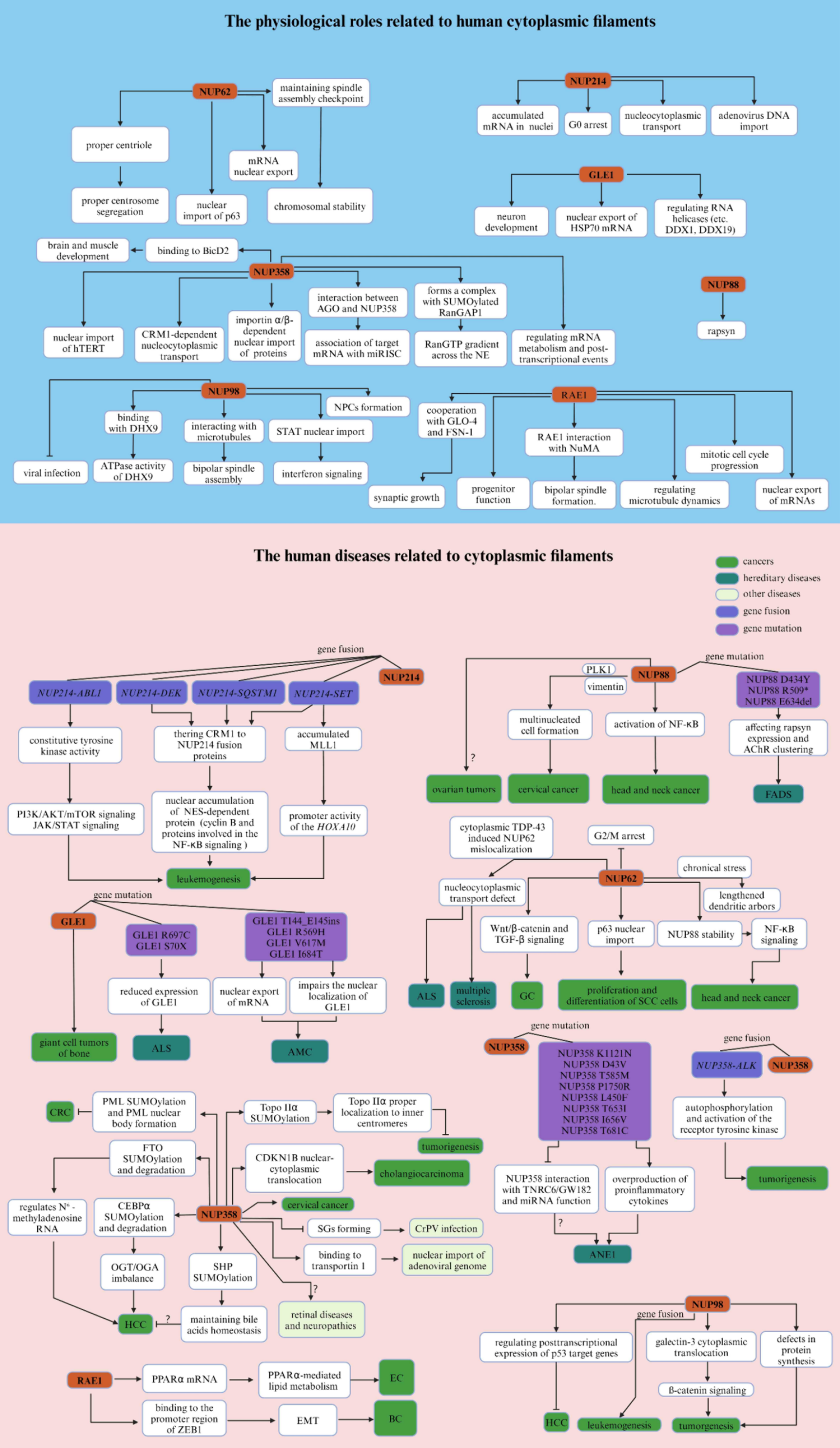
Summary of the physiological and pathological effects of each component of the cytoplasmic filaments in humans

In Xenopus laevis, the Nup214–Nup88–Nup62 complex, located in the CR subunit, is associated with two copies of the Y complex (Huang et al. 2020). In humans, the interaction between NUP88 and NUP214 facilitates the localization of NUP88 to NPCs (Fornerod et al. 1997; Bonnin et al. 2018), whereas the interaction between NUP88 and NUP62 contributes to stabilizing NUP88 by inhibiting the proteasomal degradation of overexpressed NUP88 (Bonnin et al. 2018; Singh et al. 2023). A previous study showed that genetic disruption of human *NUP88* results in pleiotropic developmental defects, including locomotor defects and neuromuscular junction dysregulation (Bonnin et al. 2018). Notably, biallelic mutations in nucleoporin NUP88 are a cause of a lethal fetal akinesia deformation sequence (FADS) characterized by impaired fetal movement, multiple congenital malformations, and a poor prognosis, which affects rapsyn, a vital regulator of the muscle nicotinic acetylcholine receptor at the neuromuscular junction (Bonnin et al. 2018) (Fig. 3).

The expression of NUP88 is abnormally upregulated in cancers, including ovarian tumors (Martínez et al. 1999), cervical cancer (Makise et al. 2021), head and neck cancer (Singh et al. 2023), and CRC (Zhao et al. 2010). Previous studies have demonstrated that elevated levels of NUP88 confer proliferation and migration advantages to cancer cells by sequestering NF-κB partly into the nucleus of unstimulated cells and consequently activating the NF-κB pathway at the level of nucleocytoplasmic transport (Singh et al. 2023; Takahashi et al. 2008). Reasonably, the Nup88-Nup214 complex, in coordination with CRM1, is believed to promote the nuclear translocation of NF-κB and pre-ribosomal assembly in Drosophila and human (Takahashi et al. 2008; Xylourgidis et al. 2006; Bernad et al. 2006). In addition, overexpression of NUP88 promotes the early stage of tumorigenesis by inducing multinucleated cell formation in both HeLa cells and a mouse model (Hashizume et al. 2010; Barr et al. 2004), which may be caused by the NUP88-dependent multinucleated phenotype resulting from changes in the organization of vimentin and polo-like kinase 1 (PLK1) (Makise et al. 2018; Naylor et al. 2016) (Fig. 3, Table 2).

**Table 2:** Related human diseases and pathological functions of each component of cytoplasmic filaments

Nucleoporins	Diseases	Abnormal changes	Functions	References
NUP88	FADS	Gene mutation (D434Y, R509* and E634del)	Affecting rapsyn expression and AChR clustering	Bonnin et al. (2018)
	Ovarian tumors	Upregulation	ND	Martínez et al. (1999)
	Cervical cancer	Upregulation	Promoting MMP-12 expression Promoting cell migration and invasion Inducing multinucleated cell formation	Makise et al. (2021), Hashizume et al. (2010)
	Head and neck cancer	Upregulation	Activation of NF-κB signaling Promoting cell proliferation and growth	Singh et al. (2023)
NUP214	AML Myelodysplastic syndrome	Gene fusion (*NUP214-DEK)*	Nuclear accumulation of NES-dependent protein (Cyclin B and proteins involved in the NF-κB signaling)	Sandén and Gullberg (2015), Saito et al. (2016), Ageberg et al. (2008)
	ALL	Gene fusion (*NUP214-SET*)	Promoting *HOXA10* gene expression nuclear accumulation of NES-dependent protein (Cyclin B and proteins involved in the NF-κB signaling)	Mendes and Fahrenkrog (2019), Boer et al. (1998)
	ALL	Gene fusion (*NUP214- SQSTM1*)	Accumulation of NES-dependent protein (Cyclin B and proteins involved in the NF-κB signaling)	Lavau et al. (2020), Gorello et al. (2010)
	ALL	Gene fusion (*NUP214-ABL1*)	activation of PI3K/AKT and JAK/STAT signaling	Simioni et al. (2016), Kleppe et al. (2011), Quintás-Cardama et al. (2008)
NUP62	GC	Upregulation	Activation of promoting Wnt/β-catenin and TGF-β signaling Promoting cell migration	Wang et al. (2021a)
	SCC	Upregulation	Promoting p63 nuclear import	Hazawa et al. (2018), Borlido and D'Angelo (2018)
	ALS	Mis-localization	Nucleocytoplasmic transport defect	Gasset-Rosa et al. (2019), Khalil et al. (2022)
	Multiple sclerosis	Mis-localization	Nucleocytoplasmic transport defect	Gasset-Rosa et al. (2019)
	Head and neck cancer	Upregulation	Promoting NUP88 stability Activation of NF-κB signaling Promoting cell proliferation and growth	Singh et al. (2023)
NUP358	Cervical cancer	Upregulation	Promoting cell growth, migration, and invasion	Wang et al. (2021b)
	HCC	Upregulation	Promoting OGT/OGA imbalance Regulating N (Mancini et al. 1996)-methyladenosine RNA Promoting malignant phenotypes	Liu et al. (2021c), Liu et al. (2020)
	Cholangiocarcinoma	ND	nuclear-cytoplasmic translocation of CDKN1B Promoting cell growth	Yang et al. (2017)
	Tumor (e.g., lung cancer, HCC, and sarcoma)	ND	Promoting Topo IIα SUMOylation and proper localization Inhibiting tumorigenesis	Dawlaty et al. (2008)
	CRC	ND	Promoting PML Sumoylation and PML nuclear body formation	Satow et al. (2012)
	Acid-related liver injury	ND	Promoting PML SUMOylation and bile acids homeostasis	Kim et al. (2016)
	Inflammatory myofibroblastic tumors	gene fusion (*NUP358-ALK*)	Promoting autophosphorylation and activation of the receptor tyrosine kinase and tumorgenesis	Ma et al. (2003), Mariño-Enríquez et al. (2011)
	Myeloid malignancies	Gene fusion (*NUP358-ALK*)	Promoting autophosphorylation and activation of the receptor tyrosine kinase and tumorgenesis	Röttgers et al. (2010), Murakami et al. (2018), Hergott et al. (2023)
	ANE1	Gene mutation (K1121N, D43V, T585M, P1750R, L450F, T6531, 1656V and T681C)	Overproduction of proinflammatory cytokines	Neilson et al. (2009), Jiang et al. (2022), Bashiri et al. (2020), Chew and Ngu (2020), Shen et al. (2021)
NUP98	Leukemias	Gene fusion (e.g., *NUP98-HOXA9*, *NUP98-HOXA11*,* NUP98-HOXA13*,* NUP98-HOXC11* etc*.*)	Regulating expression of fusion oncoproteins	Michmerhuizen et al. (2020), Sump and Brickner (2017)
	HCC	Downregulation	Regulating posttranscriptional expression of p53 target genes	Singer et al. (2012)
GLE1	giant cell tumors of bone	ND	ND	Fellenberg et al. (2016)
	ALS	Gene mutation (R697C and S70X)	Reduced expression of GLE1	Kaneb et al. (2015), Tan et al. (2017)
	AMC	Gene mutation (T144_E145ins, R569H, V617M and I684T)	Promoting mRNA export Impairing the nuclear localization of GLE1	Nousiainen et al. (2008), Paakkola et al. (2018), Kendirgi et al. 2005)
RAE1	EC	Upregulation	Promoting PPARα-mediated lipid metabolism and cell malignant transformation	He et al. (2023)
	BC	Upregulation	Promoting EMT-inducing factor ZEB1 expression and cell metastasis	Oh et al. (2017, 2019)

The human NUP214 protein, which possesses FG repeat domains and plays a critical role in nucleocytoplasmic transport, may serve as a docking site in receptor-mediated import of substrates across NPCs (Fichtman et al. 2019; Kraemer et al. 1994). Physiologically, NUP214 has been implicated in altering nucleocytoplasmic transport (Saito et al. 2016; Kindermann et al. 2019), cell differentiation (Mendes and Fahrenkrog 2019; Boer et al. 1998), and microbial infection (Cassany et al. 2015). For instance, a recent study revealed that nucleoporin fusions formed by *NUP214-SET* promote the accumulation of mixed-lineage leukemia 1 (MLL1), a histone methyltransferase essential for maintaining *HOXA10* gene expression (Oka et al. 2023; Cigdem et al. 2021). Additionally, NUP214 interacts with factors such as the mRNA export receptor TAP/NXF1 and exportin-1 (XPO1)/CRM1, facilitating mRNA export and leucine-rich nuclear export signal (NES)-dependent protein export, respectively (Saito et al. 2016) (Fig. 3).

In normal cells, NUP214 is a component of NPCs and is involved in nucleocytoplasmic transport and regulation of molecules entering and exiting the nucleus. However, in leukemic cells, NUP214 can be disrupted by chromosomal translocations, leading to the formation of fusion genes that encode aberrant proteins. One of the well-studied fusion genes involving *NUP214* is the *NUP214-DEK* fusion gene, resulting from a t(6;9) chromosomal translocation, which is associated with AML and myelodysplastic syndrome (Sandén and Gullberg 2015). The NUP214-DEK fusion protein can dysregulate transcription and contribute to leukemogenesis by promoting gene expression, which leads to uncontrolled cell proliferation and inhibition of differentiation and apoptosis (Saito et al. 2016; Ageberg et al. 2008). Another fusion protein involving NUP214 is NUP214-SET, which is created from a different chromosomal translocation and was identified in patients with T-acute lymphoblastic leukemia (T-ALL), implicating disparate leukemogenic driver mechanisms (Mendes and Fahrenkrog 2019). This fusion protein is known to be associated with leukemogenesis via aberrant activation of the *HOXA* gene cluster, which is crucial for the proliferation and survival of leukemic cells (Vlierberghe et al. 2008; Quentmeier et al. 2009). Specifically, chromosomal translocations in the 5' untranslated region (5'UTR) of NUP214, including NUP214-SET and NUP214-DEK, disrupt NUP214-dependent protein nuclear export by inhibiting the nuclear export receptor CRM1, which results in aberrant accumulation of CRM1 protein cargoes in the nucleus (Oka et al. 2019; Port et al. 2015). In addition, in T-ALL, the *NUP214-ABL1* fusion gene is known to be an oncogene that leads to constitutive tyrosine kinase activity and activation of several downstream signaling pathways (e.g., the PI3K/AKT/mTOR and JAK/STAT pathways) that are crucial for cell cycle progression and survival, ultimately leading to the pathological proliferation of T cells (Simioni et al. 2016; Kleppe et al. 2011; Quintás-Cardama et al. 2008). Furthermore, in ALL, the less frequent fusion *of NUP214-SQSTM1* also impairs the interaction between NUP214 and CRM1, therefore driving leukemogenesis (Lavau et al. 2020; Gorello et al. 2010) (Fig. 3, Table 2).

Human NUP62, which is located not only in the outer ring complex but also in the central channel, is one of several NUPs that forms the main plug and plays a role in facilitating the selective transport of molecules (Borlido and D'Angelo 2018). The known physiological functions associated with Nup62 mainly include the facilitation of selective transport, such as Cyclin B in Drosophila (Okazaki et al. 2020), mRNA in mammals (Ke et al. 2019), nuclear import of p63 (Hazawa et al. 2018), stress response (Kinoshita et al. 2014), and cell division in humans (Fukuhara et al. 2006; Hashizume et al. 2013). Specifically, it plays a role in mitotic cell cycle progression by regulating centrosome segregation, centriole maturation, and spindle orientation (Hashizume et al. 2013). Interestingly, it may be involved in protein recruitment to the centrosome after nuclear breakdown (Hashizume et al. 2013). NUP62 has been implicated in the development of various diseases, including cancers (Singh et al. 2023; Wang et al. 2021a; Borlido and D'Angelo 2018), neurological disorders (Gleixner et al. 2022; Basel-Vanagaite et al. 2006; Ding and Sepehrimanesh 2021; Harrer et al. 2023), and rheumatoid arthritis (Batliwalla et al. 2005; Senécal et al. 2014). In GC, high NUP62 expression influences cell migration and epithelial–mesenchymal transition (EMT) through Wnt/β-catenin and transforming growth factor beta (TGF-β) signaling pathways (Wang et al. 2021a). In squamous cell carcinomas (SCC), depletion of NUP62 inhibits proliferation and augments the differentiation of cells by mediating p63 nuclear import (Hazawa et al. 2018; Borlido and D'Angelo 2018). Moreover, previous studies have demonstrated that NUP62 can maintain the spindle assembly checkpoint downstream of kinetochores, thereby ensuring maintenance of chromosomal stability (Hashizume et al. 2013; Chien et al. 2020), indicating that NUP62 may participate in another potential mechanism of human cancer development. Emerging research suggests that NUP62 is involved in amyotrophic lateral sclerosis (ALS) (Gasset-Rosa et al. 2019; Khalil et al. 2022) and multiple sclerosis (Gasset-Rosa et al. 2019). One common characteristic of ALS is the abnormal aggregation of proteins, including the mislocalization and aggregation of transactive response DNA-binding protein 43 (TDP-43) (Gasset-Rosa et al. 2019; Khalil et al. 2022; Gleixner et al. 2022). NUP62 has been found to interacts with TDP-43 to regulate nucleocytoplasmic transport (Gasset-Rosa et al. 2019; Gleixner et al. 2022) (Fig. 3, Table 2).

### NUP358

In mammals, Nup358 is structurally present in cytoplasmic annulate lamellae, with parallel membrane stacks closely resembling NE (Cordes et al. 1996). Human NUP358 is distinguished by its unique structure: several zinc finger motifs, a cyclophilin A homologous domain, four Ran-binding domains, and a SUMO E3 ligase domain in mammal (Prunuske et al. 2006; Wu et al. 1995; Pichler et al. 2002). NUP358 also binds to transport receptors and modulates the activity of various protein complexes via SUMOylation in mammal (Hutten et al. 2008; Engelsma et al. 2004). Research has indicated a link between NUP358 and mRNA processing bodies (P-bodies) and stress granules (SGs), which are cytoplasmic aggregates involved in mRNA turnover and stress response (Sahoo et al. 2017). NUP358 depletion disrupts P bodies and adversely affects the miRNA pathway, suggesting its potential regulatory role in mRNA surveillance and gene expression (Sahoo et al. 2017). Nup358 also appears to be a target of the host response to viral infections. For example, the compromised function of Nup358 affects SG formation, which is a defense mechanism against viral infection in Drosophila (Sadasivan et al. 2022). In particular, cricket paralysis virus (CrPV) exploits Nup358 for replication via selective degradation of Nup358 depending on the R146 residue of CrPV, linking NPC function to a viral infection strategy (Sadasivan et al. 2022). In addition, human NUP358 interacts with the cytoplasmic dynein bicaudal D cargo adaptor 2 (BicD2), which recruits the dynein machinery to the nucleus (Gibson et al. 2023). NUP358/BicD2 interaction is crucial for mitotic spindle assembly and has profound implications for brain and muscle development (Gibson et al. 2023, 2022). Moreover, the dynein adaptor BicD2 is a master regulator of selective cargo recognition for minus-end-directed transport on microtubules, binding to cargoes and linking them to dynein motors (Gibson et al. 2023).

NUP358, as an adapter, possesses a largely disordered C-terminal region with FG repeats for gel-like phase formation and selective transport and binding sites for Ran GTPase-activating protein, Ran, and other effectors (Knockenhauer and Schwartz 2016; Frey et al. 2006; Lemke 2016; Wu et al. 1995). In mammal, Nup358 has diverse functions involved in the regulation of nucleocytoplasmic transport, viral infection, and cancer progression (Jiang et al. 2022; Gloerich et al. 2011; Wang et al. 2018), notably resulting from the combination of the intrinsic properties of its protein domains (e.g., SUMO-interacting motifs) and the various subcellular localizations of Nup358 (e.g., NPCs and kinetochores) (Liu et al. 2020; Joseph et al. 2004; Forler et al. 2004; Cooper et al. 2005). Notably, NUP358 forms a complex with SUMOylated RanGAP1, a GTPase-activating protein that catalyzes the conversion of RanGTP to RanGDP, consequently establishing a RanGTP gradient across the NE and regulating the directionality and efficiency of the nucleocytoplasmic transport processes (Wu et al. 1995; Carlon-Andres et al. 2020). NUP358 acts as a cargo- and receptor-specific assembly platform, facilitating the efficient nuclear import of proteins, such as importinα/β-cargo deleted in breast cancer 1 (DBC-1) and DNA methyltransferase 1 associated protein 1 (DMAP-1), in a receptor-independent manner (Wälde et al. 2012). In addition, NUP358 interacts with cytoplasmic human telomerase reverse transcriptase (hTERT), which is bound to NPCs by the import receptor importin 7, thereby mediating its nuclear import (Frohnert et al. 2014). Intriguingly, NUP358 is also involved in GTP hydrolysis on Ran, allowing RanGDP and importin7 to be transported to the cytoplasm where they are kept near NPCs to initiate a new round of import (Frohnert et al. 2014). In addition to facilitating protein transport, recent studies have shown that NUP358 facilitates adenoviral genome import in a manner dependent on the transport receptor transportin1 (Carlon-Andres et al. 2020). In addition, it plays a role in mRNA metabolism and post-transcriptional events, including translation (Gong et al. 2023), export (Dawlaty et al. 2008; Culjkovic-Kraljacic et al. 2012), and mRNA stability and localization regulation in humans (Gong et al. 2023; Moreno-Oñate et al. 2020). Previous studies have demonstrated that the regions of NUP358 that participate in eukaryotic translation initiation factor 4E (eIF4E)-dependent and bulk mRNA export are associated with zinc fingers and leucine-rich domains, respectively (Dawlaty et al. 2008; Culjkovic-Kraljacic et al. 2012) (Fig. 3).

Mutations and dysfunction in NUP358 can be implicated in various diseases, including retinal diseases (Castagnet et al. 2003), neuropathies (Khalaf et al. 2019; Vyas et al. 2013), cancer (Wang et al. 2021b; Yang et al. 2017), viral infections (Jiang et al. 2022), and autoimmune disorders (Senécal et al. 2014). A previous study demonstrated that the cyclophilin-like domain of NUP358 associated with the S1 subunit selectively promotes the accumulation of properly folded proteins targeted for degradation through the ubiquitin-proteasome system (UPS) (Yi et al. 2007). Notably, regulation of the UPS is thought to underlie the molecular pathogenesis of retinal aging (Kapphahn et al. 2007). Similarly, the Ran-binding domains of NUP358 that interact with Ran-GTP are linked to an age-related increase in proteasome-related proteins in the retina and the degeneration of cone photoreceptor structures (Patil et al. 2019). Retinitis pigmentosa GTPase regulator interacting protein 1 (RPGRIP1) is a critical component of cone and rod photoreceptor cells and localizes to particular foci around NPCs, suggesting its role in nuclear transport (Castagnet et al. 2003; Roepman et al. 2005). Moreover, NUP358 mediates the nucleocytoplasmic shuttling of RPGRIP1s and their interaction with other partners in amacrine and 661 W neurons, indicating its role in the pathogenesis of the retina (Castagnet et al. 2003). Neurologically, NUP358 also associates selectively with the axon initial segment (AIS) of mature neurons through interactions with ankyrin-G, an essential AIS scaffold protein (Khalaf et al. 2019). Additionally, NUP358 interacts with dishevelled and atypical protein kinase C through its N-terminal region, which is crucial for neuronal polarization during cell migration (Vyas et al. 2013) (Fig. 3, Table 2).

The conflicting roles of NUP358, a tumor suppressor and carcinogenic protein, in cancer have been previously reported. It has been reported that NUP358 is upregulated in cervical cancer cells, which potentiates the growth, migration, and invasion of cervical cancer cells (Wang et al. 2021b). In HCC, NUP358 directly promotes CCAAT/enhancer-binding protein alpha (CEBPα) SUMOylation and degradation, affecting the balance of the O-GlcNAcylation enzymes O-GlcNAc transferase (OGT) and O-GlcNAcase (OGA), subsequently triggering more O-GlcNAcylation events for oncogenic proteins (Liu et al. 2021c). Moreover, NUP358 contributes to obesity-associated protein (FTO) SUMOylation at K216, which promotes FTO degradation and subsequently promotes HCC tumorigenesis (Liu et al. 2020). Similarly, NUP358 regulates cyclin-dependent kinase inhibitor 1 B (CDKN1B) nuclear-cytoplasmic translocation by promoting CDKN1B SUMOylation at K73, thereby facilitating cholangiocarcinoma cell proliferation (Yang et al. 2017). In contrast, NUP358 suppresses CIN and tumorigenesis by maintaining its proper localization to the inner centromeres of Topo IIα during mitosis via SUMOylation (Dawlaty et al. 2008). Furthermore, NUP358 facilitates SUMOylation of promyelocytic leukemia protein (PML) transcript variant IV, favoring PML nuclear body formation and PML-IV function, and therefore plays a tumor suppressive role in CRC cells (Satow et al. 2012). Similarly, NUP358 interacts with a small heterodimer partner (SHP) at NE and mediates its SUMOylation at K68, consequently maintaining bile acid homeostasis and preventing bile acid-related liver injury, suggesting its inhibition of HCC tumorigenesis in the early stage (Kim et al. 2016). Interestingly, Nup358 interacts with P-element-induced wimpy testis (Piwi) and promotes Piwi entry into the nucleus, thereby silencing transposons and regulating genomic instability in Drosophila (Munafò et al. 2021), which could reveal its contradictory effects on cancers. In addition, *NUP358* has been found to have gene fusions (*NUP358-ALK*) in some cancers, such as inflammatory myofibroblastic tumors (Ma et al. 2003; Mariño-Enríquez et al. 2011) and myeloid malignancies (Röttgers et al. 2010; Murakami et al. 2018; Hergott et al. 2023). The *NUP358-ALK* fusion occurs because of a rearrangement within chromosome (chr) 2q13, resulting in constitutive, ligand-independent autophosphorylation and activation of the receptor tyrosine kinase ALK, which promotes uncontrolled cell growth and leads to tumor formation (Ma et al. 2003; Mariño-Enríquez et al. 2011; Lee et al. 2017) (Fig. 3 and Table 2).

To date, these mutations in *NUP358* (c.3363G>T, p.K1121N; c.128A>T, p.D43V; c.5249C>G, p.P1750R; c.1754C>T, p.T585M; c.1350A>T, p.L450F) have been identified and are believed to contribute to the development of acute necrotizing encephalopathy type 1 (ANE1) (Neilson et al. 2009; Jiang et al. 2022; Bashiri et al. 2020; Chew and Ngu 2020). Mechanistically, these mutations could cause overproduction of proinflammatory cytokines, including interleukin 6 (IL6) and tumor necrosis factor-α (TNF-α), which are associated with ANE1 (Shen et al. 2021). Specifically, individuals with missense mutations in the N-terminal region of NUP358 (mainly T585M, T653I, I656V, and T681C) secrete excessive amounts of cytokines, leading to a cytokine storm in response to viral infections, which causes neuropathology, seizures, coma, and even mortality (Shen et al. 2021). Notably, these ANE1-associated mutations impair the interaction of ANE1 with TNRC6/GW182 proteins and impair miRNA function, which may contribute to the development of ANE1 (Shen et al. 2021; Deshmukh et al. 2021) (Fig. 3, Table 2).

### NUP98

The human *NUP98* gene encodes a 186 kDa precursor that undergoes autoproteolytic cleavage to generate NUP98 and NUP96 (Fontoura et al. 1999). One of the distinctive features of NUP98 is that it can undergo a process known as "autoproteolysis," indicating that it can self-cleave into two functionally distinct parts (Sun and Guo 2008), underscoring the complex function of NUP98 within the cell. The N-terminal portion is involved in the formation of NPCs (Chatel et al. 2012), whereas the C-terminal portion is involved in gene regulation and chromatin structure (Cross and Powers 2011). Physiologically, NUP98 participates in bidirectional transport across NPCs (Miorin et al. 2020), regulates gene expression in cooperation with DExH-Box helicase 9 (DHX9) (Capitanio et al. 2017), and restricts viral infection (Nunzio et al. 2013). Alterations in NUP98, including translocations and mutations, have been associated with various forms of leukemia and other cancers (Michmerhuizen et al. 2020). *NUP98* fusion with various partners due to chromosomal translocations can lead to aberrant gene regulatory mechanisms, thereby contributing to oncogenesis.

*NUP98* gene fusions are notable abnormalities associated with various hematologic malignancies, particularly AML, CML, MLL, and myelodysplastic syndromes (Struski et al. 2017; Shima et al. 2017; Hatano et al. 1999). The N-terminal portion of NUP98 and the C-terminal portion of its fusion partner are universally involved in NUP98 fusion oncoproteins (Michmerhuizen et al. 2020). Previous studies have summarized the role of NUP98 fusion in hematologic malignancies (Michmerhuizen et al. 2020; Sump and Brickner 2017). Similarly, NUP98 exerts a carcinogenic effect by promoting galectin-3 cytoplasmic translocation (Funasaka et al. 2013) and regulating protein synthesis (Pulianmackal et al. 2022). Although the oncogenic properties of NUP98 fusion proteins in hematologic malignancies are well characterized, NUP98 plays an opposite role in other cancers. In HCC, NUP98 regulates the post-transcriptional expression of select p53 target genes and exerts its cancer-inhibitory effects (Singer et al. 2012) (Fig. 3, Table 2).

### GLE1

In human, GLE1 is a protein that shuttles between the nucleus and the cytoplasm and is known to regulate ATP-dependent DEAD-box RNA helicases (e.g., DDX1 and DDX19) in the cytoplasm; these proteins are involved in mRNA export, translation initiation, transcription termination, and stress granule dynamics (Alcázar-Román et al. 2010; Suntharalingam et al. 2004; Sharma and Wente 2020; Gray et al. 2022; Kendirgi et al. 2003) (258). Both human GLE1 isoforms exhibit diffuse cytoplasmic localization, but GLE1b is also localized to NE and NPCs (Rayala et al. 2004). In terms of transportation, GLE1b forms a heterotrimeric complex with Nup155 and CG1 in vitro and is required for the export of 70-kDa heat shock protein (HSP70) mRNA from the nucleus to the cytoplasm (Kendirgi et al. 2005, 2003). A previous study revealed that GLE1 aggregates through its N-terminal structural domain (a coiled-coil domain and a 10-amino acid aggregation-prone region) in a phosphorylation-dependent manner, and that the ability of GLE1 to self-polymerize is essential for it to perform multiple functions (Mason and Wente 2020). Interestingly, the coiled-coil structural domain and aggregation-prone region are additive for efficient mRNA export and stress granule formation, whereas the two self-polymerizing domains are independently required for the regulation of translation under cellular stress in the case of polymerization between GLE1a and GLE1b isoforms (Mason and Wente 2020). In contrast, GLE1 self-polymerization is not required for GLE1 phosphorylation or translation initiation under nonstress conditions (Mason and Wente 2020). Therefore, the polymerization state and localization of GLE1 endow it with diverse functions.

Mutations in *GLE1* cause two recessive subtypes of arthrogryposis multiplex congenita (AMC), a condition characterized by joint contractures at birth (Nousiainen et al. 2008). The two AMC subtypes related to GLE1 are lethal congenital contracture syndrome 1 (LCCS1) and lethal arthrogryposis with anterior horn cell disease (LAAHD) (Nousiainen et al. 2008). LCCS1 is a severe autosomal recessive fetal motor neuron disease that leads to severe congenital contractures, which are joint deformities present at birth and often result in prenatal or early postnatal death (Nousiainen et al. 2008; Seytanoglu et al. 2016; Whittle et al. 2021). Individuals with LAAHD present with a similar, albeit milder, phenotype with diminished fetal mobility and contractures prenatally and postnatal respiratory failure, which can result in perinatal death (Vuopala and Herva 1994). The key difference in survival between LCCS1 and LAAHD may be due to the nature of *GLE1* mutations and the resulting residual function of the GLE1 protein. The *GLE1* mutations in the two AMC subtypes (c.432-10A >G, p.T144_E145ins; c.1706G4A, p.R569H; c.1849G4A, p.V617M; c.2051T4C, p.I684T) involve the coiled-coil domain and CG1-binding domain (Nousiainen et al. 2008; Paakkola et al. 2018). The T144_E145ins mutation in *GLE1* is believed to abolish GLE1 oligomerization and shuttling, which disrupts the efficient nuclear export of mRNA at NPCs (Folkmann et al. 2013). Furthermore, other mutations (c.1706G4A, p.R569H; c.1849G4A, p.V617M; c.2051T4C, p.I684T) localized in the CG1-binding domain that interacts with CG1 to promote mRNA export (Kendirgi et al. 2005). Notably, the homozygous I684T mutation impaired the nuclear localization of GLE1, confirming its pathogenic role (Paakkola et al. 2018). Given that a previous study found that Gle1 plays a role in Schwann cells development (Seytanoglu et al. 2016), mutations in human *GLE1* may also lead to defects in neuronal development. GLE1 is involved in the cellular response to stress through SGs, which temporarily store mRNAs when cells are under stress (Aditi et al. 2019, 2015). Presumably, mutations in *GLE1* could impair this process, increasing the vulnerability of cells, particularly neurons, to stress and potentially contributing to the pathogenesis of AMC. Recently, the phenotypic spectrum of GLE1-related disorders has been extended to ALS and a mild congenital form resembling congenital myopathy (Kaneb et al. 2015; Tan et al. 2017). In addition, *GLE1* may play a crucial role in the molecular etiology of giant cell tumors of the bone through direct interaction with *miR-376a-3p* (Fellenberg et al. 2016) (Fig. 3, Table 2).

### RAE1

In the nucleus, human RAE1 interacts with other NUPs and mRNA export factors to facilitate the efficient export of mRNA molecules (Zheng et al. 2019; Addetia et al. 2021; Ren et al. 2010). Specifically, RAE1 directly contributes to the nuclear export of mRNAs by anchoring to a specific NUP98 motif (GLEBS-like motif) in NPCs (Ren et al. 2010; Pritchard et al. 1999). However, RAE1 can directly interact with other proteins (e.g., nuclear mitotic apparatus (NuMA), structural maintenance of chromosome protein 1 (SMC1), and importin β) not only in the nucleus but also in the cytoplasm, playing a critical role in mitotic bipolar spindle formation and assembly (Wong et al. 2006; Wong and Blobel 2008; Blower et al. 2005). In addition, RAE1 acts as a downstream regulatory target of the Hippo/SWH signaling pathway to promote mitotic cell cycle progression (Jahanshahi et al. 2016). Intriguingly, when the Hippo/SWH signaling pathway is inactive, RAE1 acts independently of the transcriptional coactivator Yorkie (*Yki*) gene to increase organ size by promoting mitotic S-phase entry and increasing cellular proliferation (Jahanshahi et al. 2016). When the Hippo/SWH signaling pathway is activated, it inhibits the activity of RAE1 in a WTS-dependent manner to restrict organ growth, suggesting its ability to regulate pathway homeostasis (Jahanshahi et al. 2016). Moreover, Drosophila Rae1 regulates synaptic growth in postmitotic motor neurons by binding to the evolutionarily conserved highwire (Hiw) and protecting Hiw from autophagy-mediated degradation (Tian et al. 2011). Similarly, another study showed that Rae-1 loss-of-function mutations cause axon and synapse defects by disrupting cooperation between GLO-4 and FSN-1 in Caenorhabditis elegans (Grill et al. 2012). Furthermore, one study showed that RAE1 and NUP98 maintain progenitor function through HDAC-dependent chromatin targeting to escape nucleolar localization (Neely et al. 2023).

Given the role of RAE1 in mRNA transport and spindle assembly, alterations in its expression or function may contribute to the genomic instability observed in cancer cells. There is evidence to suggest that RAE1, through its interaction with NuMA and the mitotic checkpoint protein Bub3, plays a role in maintaining proper chromosome segregation during mitosis, and disruptions in this process can lead to aneuploidy, a characteristic of many tumors (Wong et al. 2006; Stockum et al. 2018; Babu et al. 2003; Jeganathan et al. 2006; Funasaka et al. 2011). Therefore, in terms of maintaining genomic stability, RAE1 exerts tumor-suppressive effects. However, other studies have shown that RAE1 promotes cancer progression by regulating peroxisome proliferator-activated receptor alpha (PPARα)-mediated lipid metabolism (He et al. 2023) and regulating EMT signals (Oh et al. 2017, 2019). During viral infection, the matrix protein of vesicular stomatitis virus inhibits nuclear export of host cell mRNAs by binding to the mRNA export factor RAE1 (Quan et al. 2014). Similarly, ORF6, one of the proteins encoded by severe acute respiratory syndrome coronavirus 2 (SARS-CoV-2), reduced interactions with NUP98-RAE1 and consequently impaired immune evasion (Kehrer et al. 2023) (Fig. 3, Table 2).

## Inner Ring Complex

In yeast, a core feature of IR is the Z-shaped Nup188-Nup192 complex located in the middle layer, which is flanked by two roughly parallel rhomboidal structures in the inner and outer layers (Li et al. 2022). Nup188, Nup192, and Nic96 are pivotal for linking all subunits within the inner ring (IR) monomer, contributing to its stability in yeast (Li et al. 2022). At the heart of the IR subunit, a homodimer of Nup205 is central, with a pair of Nup188 molecules situated on either side of Xenopus laevis (Huang et al. 2022). This arrangement is further stabilized by four Nup93 molecules, each inserting an extended helix into the axial groove of either Nup205 or Nup188, forming part of the central scaffold of NPCs (Huang et al. 2022). Channel Nups comprising a heterotrimer of Nup62/58/54 are anchored to this central scaffold, which is critical for the function of NPC in nuclear transport in Xenopus laevis (Huang et al. 2022). In addition, six Nup155 molecules interacted with the central scaffold (Huang et al. 2022). Together with NDC1-ALADIN heterodimers, they serve to anchor the IR subunit to both the NE and outer rings of the NPCs (Huang et al. 2022).

### NUP205

The physiological roles of human NUP205 include regulating cell division by modulating mitotic timing (Bao et al. 2019), enhancing viral gene expression and replication (Lu et al. 2014; Pashkov et al. 2022), and contributing to podocyte homeostasis through the localization and activity of YAP and TAZ proteins (Ester et al. 2023). Additionally, NUP205 facilitates proper cilia function (Marquez et al. 2021; Zhang et al. 2020a), and its expression is predominantly found in the testis and cancer cells, suggesting that it may be an oncogenic marker (Fujitomo et al. 2012). Specifications: c.5984T>C (p. P1995S) disrupts the NUP205-NUP93 interaction, reducing the presence of NUP205 in NPCs (Braun et al. 2016). Together with NUP93 and XPO5, NUP205 modulates mothers against decapentaplegic homolog 4 (SMAD4), which is central to the TGF-β signaling pathway implicated in podocyte damage and SRNS (Ester et al. 2023; Braun et al. 2016; Song et al. 2022; Rahbar Saadat et al. 2020). The nuclear import of the crucial TFs YAP and TAZ mediated by NUP205 in podocytes highlights its potential involvement in the pathogenesis of SRNS through the Hippo pathway (Ester et al. 2023).

**Fig. 4:**
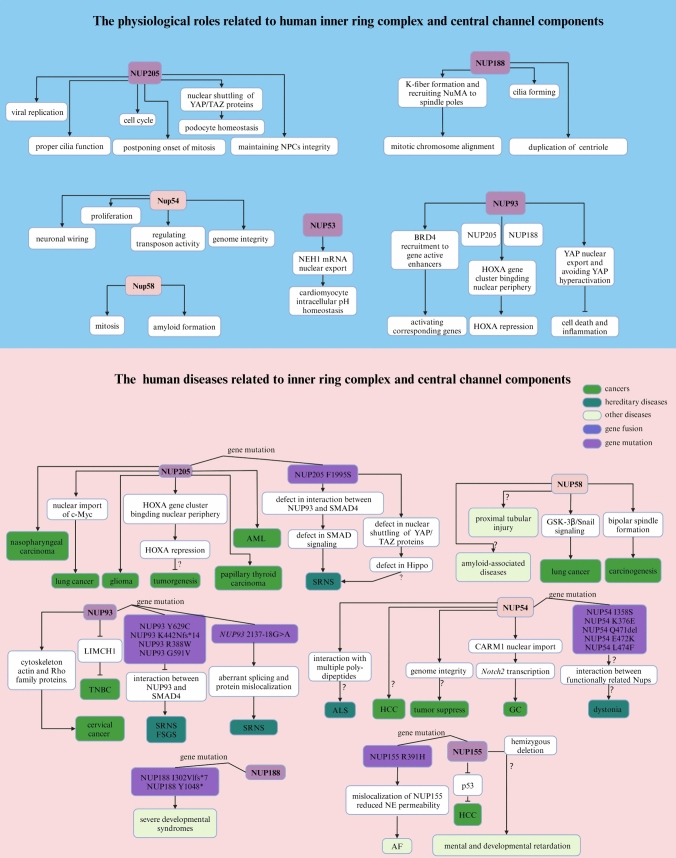
Summary of the physiological and pathological effects of each component of the inner ring complex and the central channel in humans

In gliomass, elevated NUP205 expression is correlated with reduced patient survival (Liang et al. 2023). In CRC, *NUP93*, *NUP188* and *NUP205*, are enriched at *HOXA* promoters, and their overexpression is associated with leukemia proliferation, invasion, and metastasis (Paço et al. 2020; Abuhantash et al. 2021; Labade et al. 2016). Furthermore, NUP205 has been shown to promote cell growth in lung cancer through its interaction with transmembrane protein 209 (TMEM209), which stabilizes NUP205 and regulates c-Myc nuclear entry (Fujitomo et al. 2012). Additional studies have implicated NUP205 in the progression and oncogenesis of nasopharyngeal carcinoma (Yue et al. 2020), AML (Bao et al. 2019), and papillary thyroid carcinoma (Xia et al. 2020) (Fig. 4, Table 3).

**Table 3:** Related human diseases and pathological functions of each component of the inner ring complex and central channel

Nucleoporins	Diseases	Abnormal changes	Functions	References
NUP205	SRNS	Gene mutation (F1995S)	Defect in SMAD signaling	Braun et al. (2016)
	Glioma	Upregulation	Promoting cell proliferation	Liang et al. (2023)
	Leukemias	ND	Promoting cell proliferation, invasion, and metastasis	Bao et al. (2019), Paço et al. (2020), Abuhantash et al. (2021)
	CRC	ND	Inhibiting *HOXA* expression	Labade et al. (2016)
	Lung cancer	ND	Nuclear import of c-Myc promoting cell proliferation	Fujitomo et al. (2012)
	Nasopharyngeal carcinoma	upregulation	Promoting cell proliferation	Yue et al. (2020)
	Papillary thyroid carcinoma	ND	Promoting cell growth, invasion, and migration	Xia et al. (2020)
NUP188	Severe developmental syndromes	gene mutation (I302Vlfs*7 and Y1048*)	ND	Sandestig et al. (2020)
NUP155	AF	Gene mutation (I302Vlfs*7 and Y1048*)	Impairing localization of NUP155 and NE permeability	Zhang et al. (2008), Preston et al. (2018)
	HCC	Upregulation	Promoting cancer progression	Holzer et al. (2019)
NUP93	TNBC	Upregulation	Promoting actin cytoskeleton remodeling and cell proliferation	Bersini et al. (2020)
	Cervical cancer	Upregulation	Regulating cytoskeleton actin and Rho family proteins Promoting cell proliferation, migration, and invasion	Ouyang et al. (2019)
	SRNS FSGS	Gene mutation (Y629C, K442Nfs*14, R388W and G591V)	Inhibiting interaction betweenNUP93 and SMAD4	Braun et al. (2016), Bierzynska et al. (2022), Dhanorkar et al. (2023), Hashimoto et al. (2019), Wasilewska et al. (2023)
	SRNS	Gene mutation (2137-18G>A)	Aberrant splicing and protein mis-localization	Rossanti et al. (2019)
NUP58	Lung cancer	upregulation	Activation of GSK-3β/Snail signaling promoting cell metastasis and invasion	Shi et al. (2019)
NUP54	Dystonia	Gene mutation (1358S, K376E, 0471del, E472K and L474F)	ND	Harrer et al. (2023)
	ALS	ND	Interaction with multiple poly-dipeptides	Shi et al. (2017), Khosravi et al. (2017)
	HCC	Loss of heterozygosity	ND	Huang et al. (2010)
	GC	ND	Promoting CARM1 nuclear import and cell proliferation	Wang et al. (2022)

### NUP188

Research on human NUP188 has revealed its multifaceted roles in cellular function and development, with implications for the understanding of human diseases. In Drosophila models, knockout of *Nup188* has been shown to cause motor deficits and seizure susceptibility, partially mirroring the neurological symptoms observed in humans with NUP188-related conditions (Muir et al. 2020). In addition, this phenotype is further characterized by abnormal dendrite tiling, highlighting its potential role for NUP188 in the development of dendritic structures (Muir et al. 2020). Interestingly, another study revealed that knockdown of human *NUP188* or its binding partner *NUP93* results in the loss of cilia during embryonic development, while NPC function remains largely unaffected (Viso et al. 2016). Additionally, NUP188 has been implicated in centriole duplication, a key event in cell division. NUP188 surrounds centrioles at cilia bases, functions as a component of the pericentriolar material, and is necessary for the proper duplication of centrioles, which has implications for understanding the mechanisms underlying congenital heart diseases (Viso et al. 2016; Vishnoi et al. 2020). The direct interaction between NUP188 and the centrosomal protein 152 kDa (Cep152), as well as its role in functioning at or upstream of the spikele assembly abnormal protein 6 homolog (Sas6), underscores its essential role in cell division (Vishnoi et al. 2020). NUP188 also contributes to mitotic chromosome alignment by promoting K-fiber formation and recruiting NuMA to the spindle poles, which is crucial for accurate chromosome segregation (Itoh et al. 2013). While investigating the Nup93 complex in Xenopus laevis, which includes Nup188 and Nup205, researchers found that these Nups are not essential for NPC formation (Theerthagiri et al. 2010). However, nuclei lacking Nup188 grew several-fold in size compared to those of the wild type (Theerthagiri et al. 2010). This dramatic change is due to the accelerated translocation of integral membrane proteins through NPCs, suggesting that Nup188 restricts the passage of these proteins, thereby maintaining NE homeostasis (Theerthagiri et al. 2010). Clinically, severe developmental syndromes are characterized by hypotonia, congenital cataracts, microcephaly, abnormal brain imaging, and early death due to respiratory failure, usually within the first year after birth, and are caused by homozygous nonsense mutations in the human *NUP188* gene (Sandestig et al. 2020) (Fig. 4, Table 3).

### NUP155

In Xenopus, Nup155 is involved in the fusion and formation of NEs by promoting the accumulation of Nups at the nuclear periphery (Franz et al. 2005). Additionally, proper formation of NPCs requires a self-inhibitory interaction with Nup155 in Xenopus (Magistris et al. 2018). The function of Nup155 relies on its structure, which includes the β-propeller domain that anchors the protein to the NPC and the α-solenoid region, which is essential for the correct localization of INM proteins such as the lamin-B receptor and otefin in Drosophila (Busayavalasa et al. 2012). Additionally, the α-solenoid of Nup155 exhibits chromatin-binding activity, which intensifies at the end of mitosis (Busayavalasa et al. 2012). Another study revealed that the depletion of Xenopus *Nup155* in vivo resulted in the failure of nuclear lamina formation and defects in chromosome segregation at anaphase (Franz et al. 2005). Furthermore, human NUP155 may play a role in mental and developmental retardation associated with hemizygous deletions of the 5p13 region, given the genomic location of *NUP155* (Zhang et al. 1999).

Mutations in human *NUP155* have been linked to specific phenotypes including atrial fibrillation (AF), a common cardiac arrhythmia (Zhang et al. 2008; Preston et al. 2018). A homozygous mutation, R391H, in *NUP155* co-segregates with AF and affects the nuclear localization of NUP155, reducing NE permeability (Zhang et al. 2008). Interestingly, another study demonstrated that the lamin A/C mutation p. R399C weakens the interaction between lamin A/C and NUP155, leading to AF (Han et al. 2019). This finding suggests that NUP155 functions upstream of AF at the molecular level during the pathogenesis of AF. Additionally, NUP155 is a p53 repression target that regulates p21 mRNA translation in HCC (Holzer et al. 2019) (Fig. 4 and Table 3).

### NUP93

In the assembly and function of NPCs, Nup93 functions by non-selectively binding to various Nup62-containing heterotrimers (Madheshiya et al. 2022). In addition, the conserved amino-terminal domain of Nup93 is crucial for anchoring the central channel to NPCs (Sonawane et al. 2020). In humans, the interaction of NUP93 with CCCTC-binding factor (CTCF) influences the spatial and temporal dynamics of the *HOXA* locus during cellular differentiation, indicating its role in gene regulation (Labade et al. 2021). Notably, the repression of *HOXA* by NUP93 was aided by its interacting partners NUP188 and NUP205 (Labade et al. 2016). Additionally, another study revealed that NUP93 directly activates genes with high levels of RNA polymerase II loading and transcriptional elongation by recruiting BRD4 to their active enhancers (Zhu et al. 2022). In cardiovascular research, *NUP93* deficiency has been linked to impaired endothelial NPC transport, leading to nuclear retention of YAP and subsequent exacerbation of YAP transcription, while exacerbating hypoxia-induced cardiomyocyte death and inflammation (Nguyen et al. 2024; Pan et al. 2023). In addition, Nup93 can indirectly promote IRF3 nuclear translocation by enhancing serine/threonine-protein kinase TBK1 activity and regulating antiviral innate immunity in mice (Monwan et al. 2020).

In the context of BC and cervical cancer, overexpression of NUP93 has been shown to enhance cancer growth by stimulating cell proliferation and remodeling of the actin cytoskeleton and Ras homology (Rho) family proteins (Bersini et al. 2020; Ouyang et al. 2019). Pathological mutations in NUP93 have been implicated in early-onset, severe kidney diseases, such as SRNS and focal segmental glomerulosclerosis (FSGS), with studies revealing its essential role in kidney cell function (Braun et al. 2016; Bierzynska et al. 2022; Dhanorkar et al. 2023; Hashimoto et al. 2019; Wasilewska et al. 2023). Mechanistically, *NUP93* mutations abrogated the interaction with SMAD4, causing aberrant SMAD signaling in SRNS (Braun et al. 2016). At the cellular level, NUP93 is ubiquitously expressed in various cell types within the human kidney, and its altered expression due to mutations could elucidate the pathogenic mechanisms of SRNS and FSGS (Hashimoto et al. 2019). Interestingly, NUP93 intronic variants (c.2137-18G>A) cause SRNS by leading to aberrant splicing and protein mislocalization, thereby expanding the clinical understanding of rare genetic variants underlying the disease (Rossanti et al. 2019) (Fig. 4, Table 3).

### NUP53

Nup53, also known as Nup35, is a pivotal component of NPCs and exhibits a high degree of evolutionary conservation across species (Chen et al. 2018b). Structurally, Nup53 is a highly conserved RNA-recognition motif (RRM) domain in mice, which is implicated in its ability to form homodimers (Handa et al. 2006). This dimerization is not only a structural feature but is also critical for membrane interactions and its functional role in NPC assembly (Vollmer et al. 2012). Intriguingly, a point mutation within the RRM domain of mouse Nup53 (p.F192L) leads to severe megacolon in mice, indicating its significant role in the pathology of degenerative myopathy (Parish et al. 2016). In addition to its structural roles, human NUP53 has been shown to selectively regulates intracellular pH homeostasis in cardiomyocytes by post-transcriptionally controlling the expression of sodium-hydrogen exchanger 1 (NHE1) (Xu et al. 2015b). This regulation underscores a novel functional dimension of NUP53 that connects NPC components to the regulation of cellular physiological processes.

Nup53 plays a critical role in maintaining NPC structure and nuclear integrity during the interphase in vertebrate cells (Eisenhardt et al. 2014). During mouse cell meiosis, Nup53 functions as a novel microtubule-associated protein that is important for the architecture of the oocyte meiotic spindle (Chen et al. 2018b). Its role is particularly crucial during rapid cellular divisions that occur during early embryogenesis, emphasizing its fundamental contribution to NE formation and function (Ródenas et al. 2009). The involvement of Nup53 in mitotic regulation is further underscored by its phosphorylation by two mitotic kinases, Cdk1p and Hrr25p, in yeast (Lusk et al. 2007). Furthermore, the interaction of Xenopus Nup53 with the transmembrane protein Ndc1 is vital for NPC assembly (Eisenhardt et al. 2014). Ndc1 and Nup53 also cooperatively regulate NPC density and nuclear size in early C. elegans embryos (Mauro et al. 2022) (Fig. 4 and Table 3).

## Central channel

FG repeats of the human NUP62 complex, which consists of NUP62, NUP58, and NUP54, are located on the IR plane of the central channel (Beck and Hurt 2017). During entry into mitosis, the human NUP62 complex dissolves through acetylation of NUP62 at K432 by the histone acetyltransferase TIP60, orchestrating the correct spindle orientation (Akbar et al. 2022). Additionally, the NUP62 complex anchors PLK1 to NE, facilitating NE breakdown (Martino et al. 2017). The FG repeat domains of FG-NUPs transiently bind to nuclear transport receptors via low-affinity interactions, allowing them to pass through NPCs alone or with bound cargo (Rexach and Blobel 1995). Specifically, the NUP62 complex mainly mediates the nuclear import of cargo with unconventional nuclear localization sequences (Chen et al. 2023). Genetically, the promoters of Nup54, Nup58, and Nup62 exhibit rapid accumulation of insertions/deletions (indexes) in Drosophila (McQuarrie et al. 2023), suggesting that they are vital for safeguarding genomic stability. Coincidently, another study showed that depletion of *NUP54*, *NUP62*, and *NUP58* increased cell sensitivity to ionizing radiation (Rodriguez-Berriguete et al. 2018).

### NUP58

Human NUP58 originates from the *nucleoporin-like protein 1* (*NUPL1*) gene and results from alternative mRNA splicing, which differs only in unstructured regions (Hu and Gerace 1998; Ishikawa et al. 1997). Physiologically, NUP58 contributes to the maintenance of proper mitosis (Hartono et al. 2019; Li et al. 2014) and amyloid formation (Danilov et al. 2021, 2023). Moreover, one study reported that, in the initial response to the reduced functionality of NUP58, cells exhibit physiological adaptation without genetic changes (Targa et al. 2021). However, the cells appeared to develop genetic adaptations that specifically addressed the primary impairment via focal amplification of *NUP58* and restoration of mutant protein expression (Targa et al. 2021) (Fig. 4).

In cancers, NUP58 regulates the glycogen synthase kinase 3 beta/zinc finger protein Snai1 (GSK-3β/Snail) signaling pathway (Shi et al. 2019) and bipolar spindle formation (Li et al. 2014), thereby exerting its carcinogenic effect. Additionally, NUP58 may be a potential target for proximal tubular injury (Tang et al. 2023). NUP58 is closely associated with amyloid formation (Danilov et al. 2021, 2023). Amyloid deposits are associated with different diseases, among which the most prominent are Aβ (Alzheimer's disease), α-synuclein (Parkinson's disease), amylin (type 2 diabetes), and huntingtin with polyQ expansion (Huntington's disease) (Iadanza et al. 2018). Indeed, a previous study demonstrated that NUP58 interacts with huntingtin fragments in a yeast 2-hybrid assay; thus, the underlying mechanism should be studied in the future (Kaltenbach et al. 2007) (Fig. 4, Table 3).

### NUP54

To date, the physiological roles of human NUP54 include regulating nuclear translocation (Wang et al. 2022), maintaining genome integrity (Rodriguez-Berriguete et al. 2018), and promoting the proliferation of pulmonary arterial smooth muscle cells (Yang et al. 2009). Additionally, *Nup54* is specifically expressed in previtellogenic oocytes in zebrafish, which may be related to its differentiation effects (Gautier et al. 2011). Furthermore, NUP54 and other central channel NUPs exhibited high expression levels in the brain, which is consistent with the critical role of NPCs in neurogenesis and neural maintenance (Thul and Lindskog 2018; Guglielmi et al. 2020) (Fig. 4).

Diseases associated with NUP54 include early onset dystonia (Harrer et al. 2023), cancers (Huang et al. 2010; Wang et al. 2022), psoriasis (Li et al. 2015), and ALS (Shi et al. 2017; Khosravi et al. 2017). Dystonia-associated patients had homozygous or compound-heterozygous missense and in-frame deletion variants in NUP54, all of which were located near the C-terminal end of protein (Harrer et al. 2023). Thus, these mutations in *NUP54* (c.1073T > G, p.I358S; c.1126A > G, p.K376E; c.1410_1412del, p.Q471del; c.1414G > A, p.E472K; c.1420C > T, p.L474F) may lead to destabilization of the interactions between functionally related NUPs and disassembly of the central channel (Harrer et al. 2023). In cancers, NUP54 induces nuclear import of coactivator-associated arginine methyltransferase 1 (CARM1) and, consequently, transcriptional activation and neurogenic locus notch homolog protein 2 (Notch2) methylation, thereby accelerating GC cell proliferation and tumorigenesis (Wang et al. 2022). However, *NUP54*-depleted cells also exhibited increased formation of chromosomal aberrations arising from replicated DNA (Rodriguez-Berriguete et al. 2018), suggesting that *Nup54* may also have a cancer suppressive effect by maintaining gene stability. Consistently, a previous study demonstrated that NUP54 mediates downstream critical protein phosphorylation through recruitment and direct interaction with PLK1, leading to NE disassembly and consequently, proper chromosome segregation (Martino et al. 2017). In addition, loss of heterozygosity in *NUP54* is associated with tumor size in patients with HCC (Huang et al. 2010). In ALS, proline:arginine (PRn) poly-dipeptides, caused by bidirectional transcription and ATG-independent translation of the expanded (GGGGCC)n repeat, bind to NUP54 and inhibit the movement of macromolecules into and out of the nucleus (Shi et al. 2017). Interestingly, the PRn poly-dipeptide did not bind to the unstructured monomeric form of FG repeats, but did bind to labile, amyloid-like FG polymers (Shi et al. 2017), indicating that the amyloid-like fibers formed by NUP54 enhance the toxicity of the PRn poly-dipeptide. However, a previous study showed that NUP54 can restore the proper nuclear localization of TDP-43 by alleviating the negative nuclear transport effects caused by another poly-dipeptide protein, poly-GA (Khosravi et al. 2017), suggesting that NUP54 has paradoxical pathological mechanisms through interactions with multiple poly-dipeptides in ALS (Fig. 4, Table 3).

## Nuclear basket

The human nuclear basket extends into the nucleoplasm, a substance within the nucleus. The main components of the nuclear basket include NUP153, TPR, NUP50, and ZC3HC1 (Beck and Hurt 2017; Appen et al. 2015; Gunkel et al. 2021). These proteins form distinct subcomplexes and create a lattice-like structure, similar to an actual basket, hanging from the nuclear side of NPCs (Nakielny et al. 1999). Functionally, the nuclear basket plays a pivotal role in the nuclear transport process, affecting both the import and export stages in human (Nakielny et al. 1999; Schertzer et al. 2023; Buffone et al. 2018). Moreover, the nuclear basket is implicated in chromatin organization and gene regulation in humans (Lelek et al. 2015; Kadota et al. 2020), suggesting that its influence extends beyond transport, and it may also participate in the spatial organization of the RNA processing machinery, thus playing a role in the quality control and sorting of RNAs destined for export to the cytoplasm (Nakielny et al. 1999; Ball et al. 2007). Notably, the nuclear basket is connected to the NPC central framework and can interact with the nuclear lamina and chromatin (Smythe et al. 2000; Cobb et al. 2016), further underscoring its multifunctional nature.

### NUP153

**Fig. 5:**
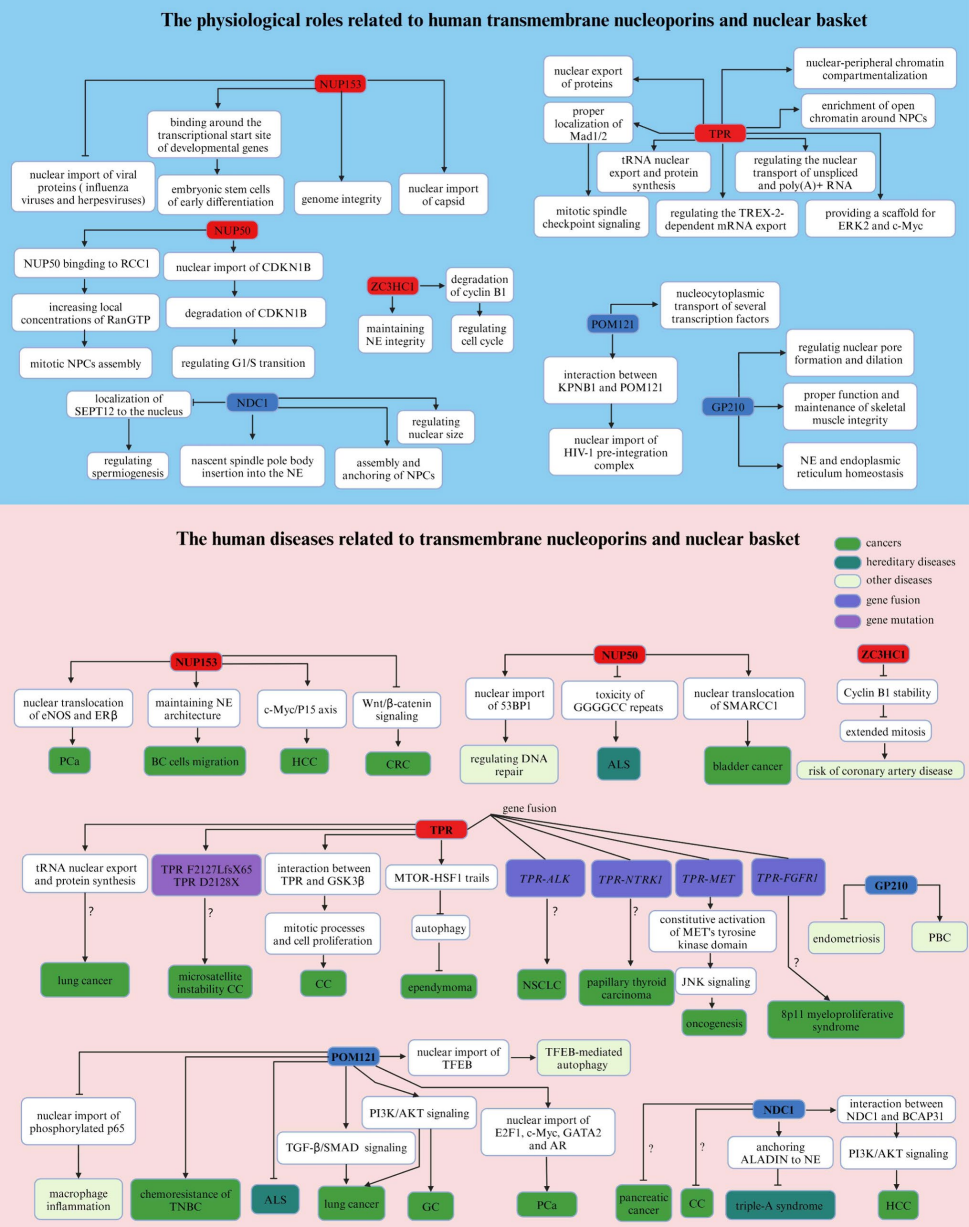
Summary of physiological and pathological effects of each component of the transmembrane nucleoporin nuclear basket in humans

In addition to being an important component of NPCs, human NUP153 is necessary for the assembly and anchoring of NPCs (Walther et al. 2001), and also acts as a docking site for the import of karyopherin (Moroianu et al. 1997). Structurally, it contains three distinct domains: an N-terminal region containing RNA-binding and pore-targeting domains (Ball et al. 2004), a central region containing multiple zinc finger motifs (Fahrenkrog et al. 2002), and a C-terminal region containing multiple FG repeats (Fahrenkrog et al. 2002). Human Nup153 has also been shown to directly interact with sentrin-specific protease 1/2 (SENP1/2) (Hang and Dasso 2002; Chow et al. 2012), importin α (Ogawa et al. 2012), importin β (Shah et al. 1998), TPR (Hase and Cordes 2003), and regulator of telomere elongation helicase 1 (RTEL1) (Schertzer et al. 2023) (Fig. 5).

Previous studies on human NUP153 have focused on nuclear transport (Shen et al. 2023a), human immunodeficiency virus (HIV) host integration (Bester et al. 2020; Xue et al. 2023), and the maintenance of genome integrity (Moudry et al. 2012; Mackay et al. 2017). Recently, growing evidence has indicated that NUP153 is closely associated with tumorigenesis. NUP153 is aberrantly expressed in various cancers, including CRC (Wu et al. 2019), HCC (Gan et al. 2022), BC (Zhou and Panté 2010), PCa (Re et al. 2018), and thyroid cancer (Ma et al. 2021). NUP153 exerts a carcinogenic effect by promoting cell motility (Zhou and Panté 2010), regulating the c-Myc/P15 axis (Gan et al. 2022), and facilitating the nuclear translocation of nitric oxide synthase 3 (eNOS) and estrogen receptor beta (ERβ) (Re et al. 2018). However, the overexpression of NUP153 negatively regulates Wnt/β-catenin signaling and suppresses the proliferation of CRC cells (Wu et al. 2019). Moreover, a previous study revealed that enhancer-specific chromatin structure and organization regulation by mammalian NUP153 is another underlying mechanism of cancer (Kadota et al. 2020). During viral infection, NUP153 interacts with the HIV-1 capsid protein, contributing to the entry of the intact capsid into the nucleus (Buffone et al. 2018; Shen et al. 2023a). However, in influenza viruses and herpesviruses, loss of function of NUP153 facilitates the nuclear import of viral proteins for viral DNA replication and assembly (Mühlbauer et al. 2015; Chang et al. 2012, 2015) (Fig. 5).

### TPR

Human nucleoporin TPR is a large protein of ~ 267 kDa that contains a coiled-coil N-terminal domain capable of forming homodimers and a highly post-translationally modified C-terminus (Hase et al. 2001). The N-terminal domain of TPR mediates intranuclear attachment to NUP153 (Hase and Cordes 2003; McCloskey et al. 2018), whereas its C-terminal domain facilitates nuclear import (Snow et al. 2013). Nucleoporin TPR forms the nuclear basket of NPCs and is vital for the enrichment of open chromatin around NPCs (Uhlířová et al. 2021). TPR plays multiple roles in the nucleus, including regulating the *TREX-2*-dependent mRNA export pathway (Aksenova et al. 2020; Lee et al. 2020), export of unspliced RNA (Rajanala and Nandicoori 2012), intranuclear transport of poly(A)+ RNA (Shibata et al. 2002), nuclear export sequence-dependent nuclear export of proteins (David-Watine 2011), and provision of a scaffold for extracellular signal-regulated kinase 2 (ERK2) (Vomastek et al. 2008) and c-Myc (Su et al. 2018). In addition, it participates in establishing nuclear-peripheral chromatin compartmentalization (Krull et al. 2010) and mitotic spindle checkpoint signaling during mitosis (Liu et al. 2023a; Lee et al. 2008) (Fig. 5).

Pathologically, TPR has been implicated in cancer through several types of abnormalities, including chromosomal translocations that generate fusion proteins with several receptor tyrosine kinases (Greco et al. 1997; Choi et al. 2014; Lee et al. 2021), regulation of nuclear translocation of RNAs (Kosar et al. 2021; Chen et al. 2021), and point mutations found in several types of cancer (Forbes et al. 2017; Moon et al. 2021); however, the underlying mechanism is unclear. One well-studied example is the *TPR-MET* oncogene, which arises from a chromosomal rearrangement that fuses the *TPR* gene with the *MET* proto-oncogene (Cooper et al. 1984; Soman et al. 1990; Yu et al. 2000; Liang et al. 1996), leading to the constitutive activation of the tyrosine kinase domain of MET and consequently activating the c-Jun N-terminal kinase (JNK) pathway, driving uncontrolled cell growth, and contributing to oncogenesis (Pal et al. 2017; Rodrigues et al. 1997). Similarly, other studies have reported the fusion of *TPR* with kinases, *including tropomyosin receptor kinase* (*TRK*) (Greco et al. 1992), *neurotrophic tyrosine receptor kinase 1* (*NTRK1*) (Greco et al. 1997; Russell et al. 2000; Créancier et al. 2015; Rekhi et al. 2021; Roccato et al. 2005), *fibroblast growth factor receptor 1* (*FGFR1*) (Kim et al. 2014; Malli et al. 2016; Qiu et al. 2017; Li et al. 2012), and ALK (Choi et al. 2014). TPR promotes transfer RNA (tRNA) nuclear export and protein synthesis in lung cancer cells (Chen et al. 2021), suggesting an oncogenic role in this context. In CC, TPR further supports oncogenesis by promoting mitotic processes and cell proliferation through its interaction with GSK3β (Dewi et al. 2018). TPR mutations have been identified in microsatellite instability CC, with specific examples (c.6381delT, p. F2127LfsX65 and c.6381dupT, p. D2128X), which may contribute to the development of cancer (Moon et al. 2021), indicating that these mutations could be function-enhancing. However, loss of heterozygosity and homozygous deletion of the *TPR* locus have been observed in human GC, which suggests that loss of function contributes to tumorigenesis in this context (Cunningham et al. 1997). Interestingly, TPR interacts with Mad 1/2 and dynein light chain to promote proper chromosome segregation during mitosis, highlighting its role in genomic stability (Lee et al. 2008; Nakano et al. 2010). Moreover, *TPR* depletion has been shown to induce mitotic catastrophe and increase the rate of tetraploidy and polyploidy, likely due to disruption of its interaction with Aurora A kinase (Kobayashi et al. 2015). These findings show that depending on the maintenance of genetic stability, TPR may also play a role in cancer inhibition. The TPR also plays a role in the response to cellular stress and senescence. Silencing of *TPR* elicits a senescent-like phenotype in cancer cells (David-Watine 2011). During oncogene-induced senescence, TPR is essential for both the formation and maintenance of internal senescence-associated heterochromatin foci, and this process is controlled by nuclear pore density (Boumendil et al. 2019). Intriguingly, another study showed that TPR depletion affected the level and function of the SUMO protease SENP2, thus affecting SUMOylation regulation at the nuclear pore and overall SUMOylation in the cell, which has broad implications for nuclear transport and cell regulation (David-Watine 2011). Furthermore, TPR protects cells from RNA-mediated replication stress by *regulating heat-shock transcription factor 1* (*HSF1*) mRNA trafficking, maintaining mTORC1 activity to phosphorylate Unc-51-like kinase 1 (ULK1), and preventing macroautophagy/autophagy induction in ependymoma (Kosar et al. 2021; Dewi et al. 2021). In addition to its cellular functions, TPR has been implicated in viral infection, as it is required for viral mRNA export, distinct from other basket NUPs, such as NUP153 and NUP50 (Bhat et al. 2023) (Fig. 5, Table 4).

**Table 4:** Related human diseases and pathological functions of each component of transmembrane nucleoporins and nuclear baskets

Nucleoporins	Diseases	Abnormal changes	Functions	References
NUP153	PCa	Upregulation	Promoting nuclear translocation of eNOS and ERβ	Re et al. (2018)
	BC	ND	Maintaining NE architecture Promoting cells migration	Zhou and Panté (2010)
	HCC	Upregulation	Promoting c-Myc/P15 axis and proliferation	Gan et al. (2022)
	CRC	upregulation	Inhibition of Wnt/β-catenin signaling	Wu et al. (2019)
TPR	Lung cancer	Upregulation	tRNA nuclear export and protein synthesis	Chen et al. (2021)
	CC	ND	Promoting mitotic processes and cell proliferation	Dewi et al. (2018)
	Microsatellite instability CC	Gene mutation (F2127LfsX65 and D2128X)	ND	Moon et al. (2021)
	Ependymoma	ND	inhibiting autophagy	Kosar et al. (2021), Dewi et al. (2021)
	NSCLC	Gene fusion (*TPR-ALK*)	ND	Choi et al. (2014)
	Papillary thyroid carcinoma	Gene fusion (*TPR-NTRK1*)	ND	Greco et al. (1997), Russell et al. (2000), Créancier et al. (2015), Rekhi et al. (2021), Roccato et al. (2005)
	Tumor	Gene fusion (*TPR-MET*)	Activation of MET's tyrosine kinase domain and JNK signaling	Cooper et al. (1984), Soman et al. (1990), Yu et al. (2000), Liang et al. (1996)
	8p11 myeloproliferative syndrome	Gene fusion (*TPR-FGFR1*)	ND	Kim et al. (2014), Malli et al. (2016), Qiu et al. (2017), Li et al. (2012),
NUP50	ALS	ND	Inhibiting toxicity of GGGGCC repeats	Freibaum et al. (2015)
	Bladder cancer	ND	Promoting nuclear translocation of SMARCC1 Promoting cell migration and proliferation	Wei et al. (2022)
ZC3HC1	Coronary artery disease	ND	Inhibiting Cyclin B1 stability and extending mitosis	Mega et al. (2015), Jones et al. (2016), Jafaripour et al. (2019)
POM121	Lung cancer	Upregulation	Activation of TGF-B/SMAD and PI3K/AKT signaling Promoting cell proliferation and metastasis	Guan et al. (2021), Zhang et al. (2020b)
	GC	Upregulation	Activation of PI3K/AKT signaling Promoting cell proliferation, migration and invasion	Kang et al. (2023)
	TNBC	ND	Promoting cell chemoresistance	Tang et al. (2022c)
	PCa	Upregulation	Promoting nuclear transport of oncogenic TFs and PCa-specific factors	Rodriguez-Bravo et al. (2018), Becker et al. (2022)
	ALS	Downregulation Abnormal function	Inhibiting transport of the TFs TFEB	Coyne et al. (2020), Brady et al. (2018)
GP210	Endometriosis	Upregulation	Increasing risk of endometriosis	Cipollini et al. (2019)
	PBC	Upregulation	ND	Nakamura et al. (2005), Wang et al. (2023b), Nakamura et al. (2006)
NDC1	HCC	Upregulation	Activation of PI3K/AKT signaling Promoting cell proliferation, invasion, and migration	Liu et al. (2024)
	Triple-A syndrome	ND	Anchoring ALADIN to NE	Yamazumi et al. (2009), Kind et al. (2009)

### NUP50

The human nucleoporin NUP50, also known as Npap60, is a key component of NPCs (Lindsay et al. 2002a). NUP50 is characterized by a motif with FG repeats and is known for its interaction with importin α/β, which is essential for the import of proteins containing a nuclear localization signal (NLS) (Park et al. 2016). Located on the nuclear side of NPCs, NUP50 interacts with the nuclear transporter importin α/β (Lindsay et al. 2002b). The function of NUP50 extends beyond merely chaperoning the import complex through NPC. It is also critical for disassembling the importin α/β-cargo complex and recycling importin (Ogawa et al. 2010a; Pumroy et al. 2012). The N-terminal region of NUP50 is particularly crucial for its function in NPC assembly, where it binds to the regulator of chromosome condensation 1 (RCC1) and stimulates guanine nucleotide exchange factor (GEF) activity toward Ran. This interaction is proposed to increase local concentrations of RanGTP, which could stimulate mitotic NPC assembly (Lindsay et al. 2002b; Ogawa et al. 2010b; Matsuura and Stewart 2005; Moore 2003) (483). Interestingly, the function of NUP50 in NPC assembly is sufficiently robust that excess RCC1 can compensate for *NUP50* depletion, indicating that NUP50, while essential, is not required for NPC formation (Holzer et al. 2021). In addition to its roles in nucleocytoplasmic transport and NPC assembly, Nup50 impacts various cellular processes, including cell cycle control and DNA repair. NUP50 has been identified as an interactor of CDKN1B, and disruption of this interaction can lead to cytosolic accumulation of CDKN1B and defects in cell proliferation (Müller et al. 2000). In terms of development, NUP50 is weakly expressed in most tissues but is highly expressed in the developing neural tube and adult testes (Smitherman et al. 2000; Park et al. 2016). Its deficiency can cause embryonic lethality, neural tube abnormalities, exencephaly, and intrauterine growth retardation in mice, indicating its importance in embryonic development (Smitherman et al. 2000; Park et al. 2016) (Fig. 5).

NUP50 is strongly associated with ALS pathogenesis in neurological diseases. The expansion of GGGGCC repeats in the non-coding region of C9orf72 is the most common cause of sporadic and familial forms of ALS and frontotemporal dementia (Megat et al. 2023). Loss of Nup50 enhances the toxicity of GGGGCC repeats in Drosophila (Freibaum et al. 2015). In addition, another study demonstrated that mutations in *NUP50* associated with ALS can decrease the expression of NUP50 and disrupt its normal function, linking NUP50 and ALS (Megat et al. 2023). Previous studies have shown that *NUP50* knockdown can cause cytoplasmic inclusions of nuclear pore components and RanGAP1 (Zhang et al. 2015; Chou et al. 2018), impairing the nuclear pore function. RanGAP1 is a key protein that regulates nucleocytoplasmic shuttling, and its aberrant localization and expression have been observed in ALS patients (Gasset-Rosa et al. 2019; Li et al. 2017). In addition, alterations to the nuclear pore are sufficient to cause dysfunction of TDP-43 in both human neurons and ALS patients (Coyne et al. 2021; Lek et al. 2016). In bladder cancer, NUP50 promotes the nuclear translocation of the SWI/SNF complex subunit SMARCC1, facilitating cell migration and proliferation (Wei et al. 2022). However, NUP50 can promote the recruitment of DNA double-strand break repair factor 53BP1 to DNA repair foci by antagonizing BRCA1-dependent events (Mackay et al. 2017) (Fig. 5, Table 4).

### ZC3HC1

ZC3HC1, also known as the nuclear-interacting partner of ALK (NIPA), is a protein that is encoded by the *ZC3HC1* gene on chr 7 in humans (Zhang et al. 2000). Recent findings have shown that ZC3HC1 constitutes a novel and intrinsic element of the nuclear basket ubiquitously present at the NE in a diverse array of cells, encompassing both proliferating and non-dividing terminally differentiated cell types of various morphogenetic lineages (Gunkel et al. 2021; Gunkel and Cordes 2022). In the context of its molecular function, ZC3HC1 is instrumental in the assembly of the SCF (NIPA) E3 ubiquitin ligase complex (Bassermann et al. 2005). This complex is pivotal for the temporal regulation of the cell cycle, specifically orchestrating the transition to mitosis by mediating ubiquitination and the subsequent proteasomal degradation of Cyclin B1 (Bassermann et al. 2005). The regulatory influence of ZC3HC1 extends to the maintenance of NE integrity (Gunkel et al. 2021) and is thought to contribute to broader nuclear activities (Gunkel et al. 2021; Gunkel and Cordes 2022), underscoring its versatility within the cellular environment. Pathologically, ZC3HC1 is associated with an increased risk of coronary artery disease by regulating Cyclin B1 stability (Mega et al. 2015; Jones et al. 2016; Jafaripour et al. 2019). Additionally, a single nucleotide polymorphism in *ZC3HC1* is associated with increased carotid intima-media thickness in patients with rheumatoid arthritis (López-Mejías et al. 2013; Linseman et al. 2017). Furthermore, certain *ZC3HC1* polymorphisms are involved in the incidence of hypertension (Ma et al. 2019a; Kunnas and Nikkari 2015), suggesting that this gene has a far-reaching impact on vascular health. Overall, the emerging portrait of *ZC3HC1* is that of a multifaceted protein integral to cell cycle progression and nuclear architecture, and is potentially implicated in various disease states, meriting further investigation to fully delineate its biological roles and therapeutic potential (Fig. 5, Table 4).

## Transmembrane nucleoporins

Human transmembrane nucleoporins, including POM121, GP210, and NDC1, play important roles in the selective transport of macromolecules across the NE through NPCs and carry the onus of anchoring the NPCs at the NE and ER (Eisenhardt et al. 2014; Bindra and Mishra 2021; Mansfeld et al. 2006). In addition to their transport function, human transmembrane nucleoporins are involved in multiple cell processes, including the regulating of gene expression (Franks et al. 2016), cell differentiation (Gomez-Cavazos and Hetzer 2015; Sharma et al. 2014), and NE assembly (Güttinger et al. 2009).

### POM121

POM121 is encoded by the tandem gene locus *POM121A/C* on human chromosome 7q11.23 (Funakoshi et al. 2007). Notably, this chr region is associated with the inherited developmental disease Williams–Beuren syndrome (Osborne and Mervis 2007). Structurally, POM121 contains a single-pass N-terminal membrane-spanning domain that directly binds to the β-propeller regions of NUP155 and NUP160, an NLS that interacts with importin α/β, and a C-terminal cytoplasmic/nucleoplasmic domain with FG repeats that aid cargo carriage (Mitchell et al. 2010; Funakoshi et al. 2011). Notably, specific regions within POM121, specifically the FG repeats and an adjacent α-helix structure, are critical for its role in mediating the nuclear import of HIV-1 pre-integration complex (Guo et al. 2018). In terms of transport, POM121 is known to facilitate the nucleocytoplasmic transport of several TFs, including E2F1, c-Myc, the endothelial transcription factor GATA2, proliferator-activated receptor alpha gamma (PPARγ), and AR (Rodriguez-Bravo et al. 2018; Yu et al. 2024). Interestingly, a recent study revealed that POM121 is not only a structural component of the nuclear pore, but also has a chaperone-like capacity to facilitate the signaling required for transcription factor EB (TFEB)-mediated autophagy (Wang et al. 2023c) (Figure 5).

In various cancer types, including CRC (Wang et al. 2020), lung cancer (Guan et al. 2021; Zhang et al. 2020b), laryngocarcinoma (Zhao et al. 2020), PCa (Becker et al. 2022), GC (Kang et al. 2023), and oral SCC (Ma et al. 2019b), the oncoprotein POM121 is universally overexpressed and plays a role in modulating key signaling pathways. In lung cancer (Guan et al. 2021; Zhang et al. 2020b) and GC (Kang et al. 2023), POM121 overexpression has been associated with abnormal activation of important signaling pathways, such as the TGF-β/SMAD and PI3K/AKT pathways. In addition, it promotes PCa tumor growth by enhancing the nuclear transport of oncogenic TFs, such as E2F1 and c-Myc, as well as PCa-specific factors, such as AR and GATA2 (Rodriguez-Bravo et al. 2018). Furthermore, POM121 expression increases with tumor progression, suggesting that POM121 may be a target molecule for patients with advanced PCa (Becker et al. 2022). In triple-negative breast cancer (TNBC), overexpressed POM121 facilitates cell drug resistance (Tang et al. 2022c). Regarding the immune response, POM121 has been shown to suppress macrophage inflammatory responses by selectively inhibiting the nuclear accumulation of NF-κB (Ge et al. 2019) (Fig. 5, Table 4).

In C9orf72-associated ALS, there is a reduction in the levels of POM121 and consequently other NUPs in induced pluripotent stem cell-derived neurons (Coyne et al. 2020). The reduction in POM121 was believed to result from expanded C9orf72 amyotrophic lateral sclerosis overlapping with frontotemporal dementia (ALS/FTD) repeat RNA alone (Coyne et al. 2020). This reduction appears to initiate a cascade of events leading to cellular toxicity, which may contribute to neurodegeneration observed in ALS. In addition to the aberrant expression of POM121, other studies have shown that the normal function of POM121 in the transport of TFEB (Wang et al. 2023c; Lin et al. 2024), which is vital for the autophagy-lysosome pathway (Brady et al. 2018), is impaired in C9orf72-ALS. Autophagy is a cellular process for degrading and recycling cellular components, and its dysfunction is a feature of several neurodegenerative diseases, including ALS (Chua et al. 2022). Additionally, the sigma-1 receptor (SIGMAR1) facilitates the nuclear transport of TFEB by interacting with POM121 (Wang et al. 2023c). However, pathological hexanucleotide repeat expansion disrupts this interaction in C9orf72-ALS models, leading to impaired autophagy (Wang et al. 2023c). Interventions that enhance SIGMAR1 activity or directly increase POM121 function, such as the use of SIGMAR1 agonists such as pridopidine or fluvoxamine, have shown promise in restoring normal nucleocytoplasmic transport and autophagy, and may provide therapeutic benefits for patients with ALS (Wang et al. 2023c). In summary, POM121 has been implicated in a wide range of cellular processes, from viral infection to cancer progression and neurodegeneration (Fig. 5, Table 4).

### GP210

Human GP210, a transmembrane protein, was the first nucleoporin identified and has been described as having tissue-specific expression, particularly within epithelial cells (Wozniak and Blobel 1992; Olsson et al. 1999). Its expression is not static; gp210 is dynamically regulated during cellular differentiation, as evidenced in mouse myoblasts (Sakuma et al. 2022). Although initially not expressed in these cells, gp210 expression markedly increases during the mouse myogenic differentiation process, highlighting its critical role in the proper function and maintenance of skeletal muscle integrity (Sakuma et al. 2022). Consistently, the N-terminal domain of gp210, which projects into the perinuclear space, has been identified as a mediator of mouse muscle cell differentiation in vitro, and is believed to be vital for NE and ER homeostasis (Gomez-Cavazos and Hetzer 2015). In addition to its role in muscle differentiation, GP210 is involved in fundamental processes of nuclear pore formation and dilation (Cohen et al. 2003). Additionally, gp210 is implicated in NPC disassembly and NE breakdown during embryonic cell division (Galy et al. 2008), which is pivotal for ensuring proper cell cycle progression. Interestingly, the presence of Drosophila gp210 in an abundant non-nuclear form during embryogenesis suggests that it may play a key role in facilitating the swift assembly of nuclei, which is critical during the rapid cellular division and differentiation stages of development (Berrios et al. 1995). Moreover, gp210 is dispensable for the incorporation of other Nups, such as Pom121 or Nup107, and for maintaining the stability of NPCs in mouse cell (Eriksson et al. 2004) (Fig. 5).

The relevance of human GP210 extends to its clinical applications, particularly in the context of autoimmune diseases. It has been implicated in endometriosis (Cipollini et al. 2019) and more notably, in primary biliary cirrhosis (PBC) (Nakamura et al. 2005, 2006; Wang et al. 2023b), a chronic liver condition. The detection of antibodies against GP210 is a strong indicator of PBC, and antibody titers against its C-terminal peptide serve as a clinical parameter for monitoring the disease (Nakamura et al. 2005; Wang et al. 2023b). Moreover, the heightened expression of GP210 on the NE of biliary epithelial cells in PBC patients might be associated with inflammatory damage that contributes to the autoimmune response, potentially leading to the progression of liver failure in this disease (Nakamura et al. 2006) (Fig. 5, Table 4). These insights into the functions of GP210 and its association with disease states underscore its potential as a biomarker and target for therapeutic intervention.

### NDC1

Human NDC1 is a multifunctional protein that plays a crucial role in the structural and functional organization of NE. NDC1, as a component of NPCs, plays a key role in assembly (Stavru et al. 2006) and anchoring of NPCs to the NE via interactions with NUP53 and ALADIN (Huang et al. 2022; Mansfeld et al. 2006). In addition, Ndc1 acts within the NE to mediate the insertion of the nascent spindle pole body in yeast, which is critical during cell division (Winey et al. 1993; Chen et al. 2014). Aberrations in the functions of Ndc1, such as overexpression, have been shown to cause spindle pole body duplication defects in yeast, highlighting its importance in maintaining cellular integrity (Chial et al. 1999). Moreover, loss-of-function mutations in Ndc1 can lead to severe developmental consequences, such as high mortality rates in organisms such as Caenorhabditis elegans, due to NPC defects (Stavru et al. 2006). In the broader context of cellular architecture, the role of Ndc1 extends to regulating NPC density and nuclear size in the elegans embryo, acting in concert with the Nup53 and membrane biogenesis pathways (Mauro et al. 2022). In humans, SEPT12-NDC1 complexes have been implicated in spermiogenesis in mammals (Lai et al. 2016), emphasizing the versatility of NDC1. The reach of the protein also extends to vesicle trafficking, where it has been suggested that ARRDC5 could influence the transport and localization of mouse Ndc1, alongside other proteins vital for the formation of the sperm head–tail coupling apparatus (Liu et al. 2023c). Furthermore, human NDC1's direct interaction with ALADIN is not only a structural requirement, but also critical in disease, as mutations in ALADIN that disrupt this interaction are implicated in the pathogenesis of triple-A syndrome (Yamazumi et al. 2009; Kind et al. 2009) (Fig. 5).

The relevance of NDC1 is also evident in the context of oncology. It is implicated in the promotion of tumorigenesis in HCC by enhancing the PI3K/AKT signaling pathway in cooperation with B-cell receptor-associated protein 31 (BCAP31) (Liu et al. 2024). Moreover, compared with those in normal tissues, the expression levels of NDC1 in CC tissues were significantly elevated, with higher expression levels correlating with increased patient survival (Liu et al. 2021b). Conversely, elevated NDC1 is a marker of poor prognosis in HCC (Liu et al. 2023b, 2024) and pancreatic cancer (Shen et al. 2023b), suggesting its dual role in cancer biology. In summary, NDC1 is a key player in the maintenance of cellular structure and function, with roles ranging from cell division to spermiogenesis, and from vesicle trafficking to disease pathogenesis (Fig. 5, Table 4). The diverse roles of this protein in various cancers further illustrate its importance as a focal point for both the understanding and treatment of these diseases.

## Conclusion and perspective

Human NPCs serve as critical gateways that regulate the flow of molecules between the nucleus and cytoplasm, and play a pivotal role in numerous cellular processes. Their importance extends far beyond their barrier function, as they are actively involved in gene regulation, RNA processing, and cell cycle progression. The intricate structure and function of NPCs are essential for maintaining cellular homeostasis, and disruptions in their components can lead to a range of human diseases. Cancers, for instance, have been linked to NPC dysfunction, where alterations in nucleocytoplasmic transport may contribute to the aberrant growth and division of cells (Gan et al. 2022; Nofrini et al. 2016; Fujitomo et al. 2012). Neurodegenerative conditions such as ALS/FTD have also been associated with dysregulation of human NUPs (Coyne et al. 2020; Gasset-Rosa et al. 2019; Khalil et al. 2022), indicating a broader impact of NPC integrity on neuronal health. Furthermore, pathogens, such as viruses, exploit NPCs to propagate and disrupt cellular integrity (Sadasivan et al. 2022; Quan et al. 2014). Moreover, inherited conditions such as triple-A syndrome underscore the critical nature of NPC components in human biology (Yamazumi et al. 2009; Kind et al. 2009; Osborne and Mervis 2007). The insights gained into NPCs could revolutionize our approach for the diagnosis and treatment of diseases. In addition, new NPC components, such as the newly discovered ZC3HC1, still need further discovery (Gunkel et al. 2021; Gunkel and Cordes 2022). This provides a new direction for fully understanding NPC functions. Research is increasingly focused on unraveling the complex molecular mechanisms underlying NPC functions. This could pave the way for identifying therapeutic targets within NPC assembly and transport pathways. The potential of NPCs as biomarkers is also significant, as changes in NPC function could signal the early onset of the disease or track its progression.

Currently, there are no drugs that specifically target NPC components. However, certain drugs that interfere with transport factors dependent on NPCs have been identified. One such example is importazole, a 2,4-diamino quinazoline compound that disrupts the interaction between importin β and NPCs (Soderholm et al. 2011). This disruption effectively inhibits the nuclear import of cargo proteins while leaving transportin-mediated nuclear import or CRM1-mediated nuclear export unaffected (Soderholm et al. 2011). Another compound, leptomycin B, is a natural compound that selectively binds to the nuclear export signal-binding region of CRM1 (Rahmani and Dean 2017). Leptomycin B decreases the nuclear availability of CRM1 by promoting the redistribution of CRM1 from the nucleus to the cytoplasm (Rahmani and Dean 2017). Additionally, a previous study demonstrated that 5-fluorouracil disrupts nuclear export and NPC permeability in a calcium-dependent manner, which has been associated with drug resistance (Cristoferi et al. Mar 2021). Therefore, further investigations of drugs targeting specific components of NPCs are promising for refining treatment strategies.

## Data Availability

Not applicable.
